# Accurate and Noninvasive Dysphagia Assessment via a Soft High‐Density sEMG Electrode Array Conformal to the Submental and Infrahyoid Muscles

**DOI:** 10.1002/advs.202500472

**Published:** 2025-03-24

**Authors:** Weijie Hong, Lin Mao, Kai Lin, Chongyuan Huang, Yanyan Su, Shun Zhang, Chengjun Wang, Daming Wang, Jizhou Song, Zuobing Chen

**Affiliations:** ^1^ Department of Rehabilitation Medicine The First Affiliated Hospital School of Medicine Zhejiang University Hangzhou 310003 China; ^2^ Key Laboratory of Soft Machines and Smart Devices of Zhejiang Province State Key Laboratory of Brain‐Machine Intelligence Department of Engineering Mechanics Zhejiang University Hangzhou 310027 China; ^3^ Huanjiang Laboratory Zhuji 311899 China

**Keywords:** dysphagia assessment, machine learning, stretchable high‐density sEMG, swallowing

## Abstract

Accurate, noninvasive dysphagia assessment is important for rehabilitation therapy but current clinical diagnostic methods are either invasive or subjective. Surface electromyography (sEMG) that monitors muscle activity during swallowing, offers a promising alternative. However, existing sEMG electrode arrays for dysphagia assessment remain challenging in combining the advantages of a large coverage area and strong compliance to the entire swallowing muscles. Here, we report a stretchable, breathable, large‐area high‐density sEMG (HD‐sEMG) electrode array, which enables intimate contact to complex surface of the submental and infrahyoid muscles to detect high‐fidelity HD‐sEMG signals during swallowing. The electrode array features a 64‐channel soft on‐skin sensing array for comprehensive data capture, and a stiff connector for simple and reliable connection to an external acquisition setup. Systemically experimental studies revealed the easy operability of the soft HD‐sEMG electrode array for effortless integration with the skin, as well as the excellent mechanical and electrical characteristics even subject to substantial skin deformations. By comparing HD‐sEMG signals collected from 38 participants, three objective indicators for quantitative dysphagia evaluation were discussed. Finally, a machine learning model was developed to accurately and automatically classify the severity of dysphagia, and the factors affecting the recognition accuracy of the model were discussed in depth.

## Graphical abstract

A stretchable, breathable, large‐area high‐density sEMG (HD‐sEMG) electrode array is reported, which enables seamless integration to the complex surface of the submental and infrahyoid muscles to detect high‐fidelity HD‐sEMG signals during swallowing. Assisted with a machine learning model, the severity of dysphagia can be automatically and noninvasively assessed with a high accuracy.

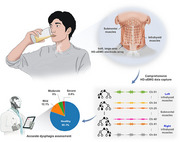

## 1 Introduction

Dysphagia, also known as swallowing disorder, refers to the difficulty or inability to swallow orally ingested food through the throat and esophagus into the stomach, which can lead to serious health risks such as aspiration pneumonia, dehydration, and malnutrition.^[^ ^1^, ^2^ ^]^ This disease has affected diverse populations around the world, especially the elderly adults and stroke survivors.^[^ ^3^, ^4^ ^]^ According to epidemiological surveys, the prevalence of dysphagia among the elderly in the general community is 30.52%, while it is as high as 58.69% in nursing institutions.^[^ ^5^ ^]^ As the population continues to age, dysphagia has become common in clinical settings, and this chronic disease that usually requires long‐term care has received widespread attention.

A normal swallowing generally includes four stages: oral preparation stage, oral propulsion stage, pharyngeal stage and esophageal stage, which are innervated by multiple muscles in sequence.^[^ ^6^, ^7^ ^]^ Among them, the submental and infrahyoid muscles play a vital role, which determines whether food can pass smoothly through the mouth and throat into the esophagus. For example, the submental muscles ensure effective control of the hyoid bone and are essential for lifting and stabilizing the tongue to push the hyoid bone forward and help move the food bolus to the entrance of the esophagus. Their contraction will initiate the apnea period during swallowing and prevent food from entering the wrong cavity, such as the airway. The suprahyoid muscles, including the thyrohyoid, sternothyroid, and sternohyoid muscles, are responsible for ensuring the safe and efficient passage of the food bolus into the esophagus through a series of coordinated activities. The contraction of the thyrohyoid muscle moves the thyroid cartilage and larynx upward toward the hyoid bone, while the sternothyroid and sternohyoid muscles pull the thyroid cartilage and hyoid bone downward. These actions also help close the vocal cords and provide proper passage for the food bolus.

To monitor the swallowing process and assess the severity of dysphagia for precise rehabilitation, a variety of evaluation methods have been employed clinically, such as the videofluoroscopic swallow study (VSS),^[^ ^8^ ^]^ the fiberoptic endoscopic evaluation of swallowing (FEES),^[^ ^9^ ^]^ and clinical scale assessments (CSA).^[^ ^10^, ^11^ ^]^ However, these traditional diagnostic methods are either invasive or subjective. As a simple, noninvasive, and objective technique for the muscle function assessment and disease diagnosis, surface electromyography (sEMG), which detects muscle activity by epidermal electrode arrays, has attracted widespread attention and been widely explored as a feasible means for the assessment of dysphagia.^[^ ^12^, ^13^, ^14^, ^15^, ^16^, ^17^ ^]^ For example, Miller et al.^[^ ^18^ ^]^ and Murakami et al.^[^ ^19^ ^]^ fabricated flexible large‐area high‐density sEMG (HD‐sEMG) electrode arrays to detect muscle activity of the submental and infrahyoid muscles during swallowing. However, these flexible HD‐sEMG electrode arrays made of a rigid polyimide (PI) substrate exhibit mechanical properties that are extremely mismatched with the soft, deformable skin, making it difficult to maintain intimate contact with the skin for capturing high‐fidelity sEMG signals under dynamic deformations. To overcome this issue, stretchable thin‐film sEMG electrodes were developed by patterning the thin PI substrate into a stretchable network structure,^[^ ^20^, ^21^, ^22^, ^23^, ^24^, ^25^, ^26^, ^27^ ^]^ or by using intrinsically stretchable soft materials,^[^ ^28^, ^29^, ^30^, ^31^, ^32^, ^33^ ^]^ which have been demonstrated as a promising solution to improve the conformality of electrodes with irregular, complex skin surfaces. However, these stretchable sEMG electrode designs have sparse electrode distribution or small coverage area, and can only cover the submental muscle or the infrahyoid muscle, making it difficult to capture the entire spatiotemporal characteristics of muscle activity during swallowing, therefore resulting in low assessment accuracy.

**Figure 1:**
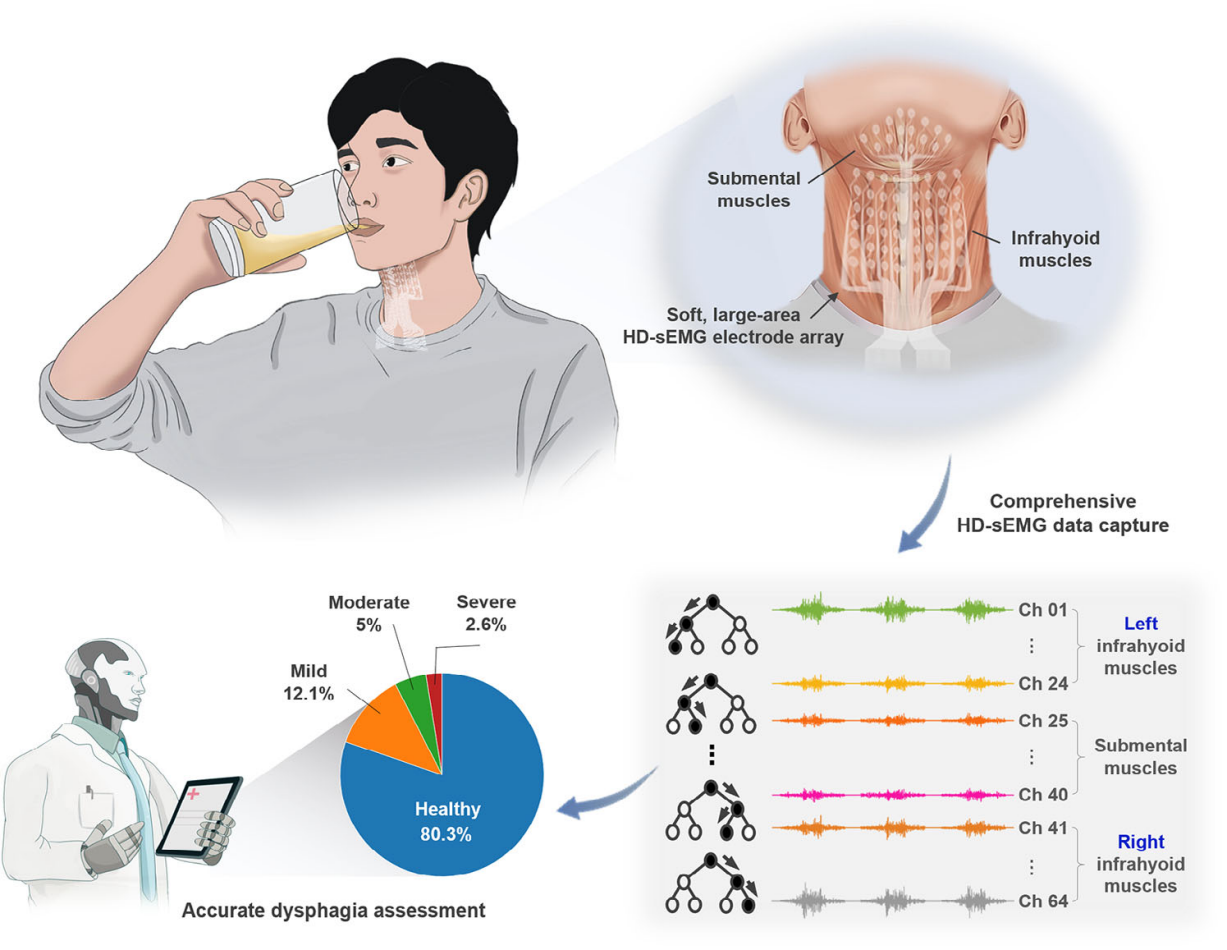
Schematic illustration of the accurate and noninvasive dysphagia assessment via a soft, large‐area HD‐sEMG electrode array conformal to the submental and infrahyoid muscles.

While there have been notable advances in sEMG based dysphagia assessments, HD‐sEMG electrode arrays that incorporates with advantages of a large coverage area and high‐density distribution, strong conformability to the entire swallowing muscles group, and easy operation remain challenging. Furthermore, appropriate dysphagia assessment methods integrating with HD‐sEMG signals from a large number of patients are also lacking. Here, we report a stretchable, large‐area HD‐sEMG electrode array, which can conformably cover the submental and infrahyoid muscles to comprehensively detect muscle activity during swallowing, as schematically illustrated in **Figure** **1**. The electrode array, which was designed via a region‐specific integration strategy and rapidly fabricated by a scalable, low‐cost screen‐printing technology, features a 64‐channel soft on‐skin sensing array for high‐fidelity data capture and a stiff flexible printed circuit (FPC) connector for simple and reliable electrical connection to an external data acquisition setup. To improve the handling operability and achieve the convenient integration with the skin, the soft large‐area sensing arrays with tissue‐soft mechanical properties and strong adhesion were sandwiched between two releasable stiff polyethylene terephthalate (PET) layers to form a rigid‐soft‐rigid composite. By comparing HD‐sEMG signals collected from 38 participants of healthy young adults, healthy elderly adults, and elderly stroke patients in nine swallowing tasks, three objective indicators including the muscle activity intensity map, the degree of symmetry, and the change of the barycenter, were obtained for quantitative evaluation of dysphagia. Assisted with a machine learning model, the severity of dysphagia can be automatically assessed with a high accuracy.

## 2 Results

### 2.1 Design and Fabrication of the Soft, Large‐Area HD‐sEMG Electrode Array

**Figure 2:**
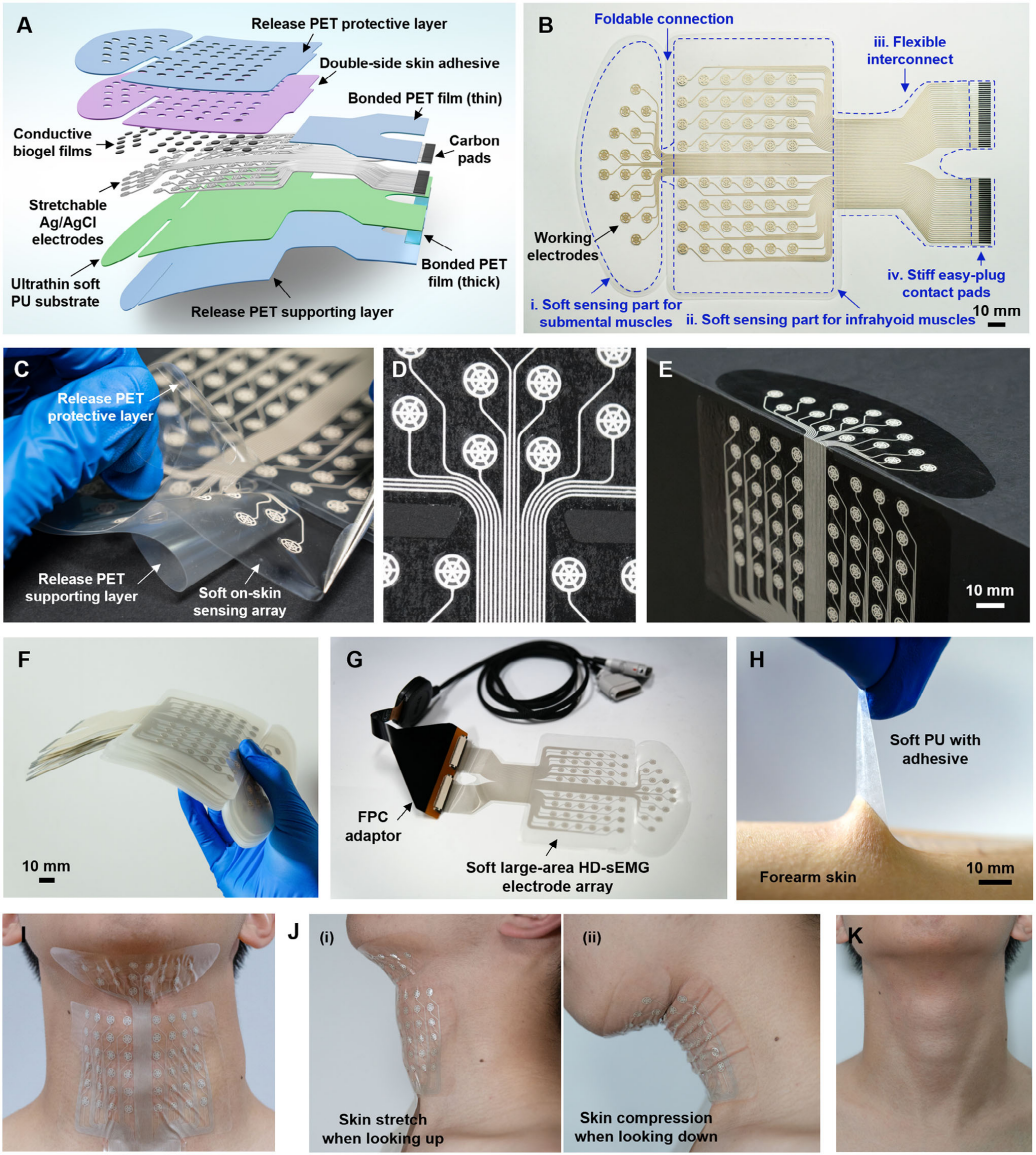
Design of the soft, large‐area HD‐sEMG electrode array for comprehensive detection of swallowing activity. A) Exploded view layout and B) optical image of the soft, large‐area HD‐sEMG electrode array. C) Photograph showing release PET supporting layer, soft electrode layer and release PET protective layer. D,E) Optical images showing the foldable connection adhered on a flat and right‐angle surface. F) Photograph of a stack of the HD‐sEMG electrode array. G) Photograph of the HD‐sEMG electrode array connected to a commercial data acquisition cable through a home‐made pluggable FPC adaptor. H) Photograph showing strong adhesion between the soft PU film with adhesive and human skin. I,J) Photograph of the HD‐sEMG electrode array adhered on both submental and infrahyoid muscles under substantial skin deformations. K) Photograph of skin after the removal of the HD‐sEMG electrode array.

**Figure** **2**A,B shows the soft large‐area HD‐sEMG electrode array for dysphagia assessment, which is functionally divided into four parts: two soft on‐skin sensing arrays (thickness, 95 µm), a flexible interconnect (thickness, 120 µm) and a stiff contact pad (thickness, 3.05 mm). The two soft sensing arrays comprise i) an ultrathin soft polyurethane (PU) layer (thickness, 50 µm) as stretchable and breathable substrate,^[^ ^21^ ^]^ ii) stretchable Ag/AgCl conductive traces (thickness, 10 µm) serving as both sensing electrodes and interconnects, iii) a biocompatible conductive biogel film^[^ ^34^ ^]^ on each electrode site to reduce the electrode‐skin interface impedance, and iv) a biocompatible acrylic double‐sided adhesive (thickness, 45 µm) to isolate the conductive traces and ensure close contact with the skin. Such a soft and thin mechanics design of the two sensing arrays is intended to ensure seamless integration with the complex epidermal surfaces of the submental and infrahyoid muscles for comprehensive detection of muscle activities during swallowing. However, its low bending rigidity also makes it prone to curling, which poses challenges in subsequent handling operations and the layer‐by‐layer fabrication. To overcome this dilemma, the two soft sensing regions are sandwiched between a release PET supporting layer (thickness, 50 µm) and a release PET protective layer (thickness, 25 µm) via van der Waals forces to form a rigid‐soft‐rigid combined construction (Figure 2C). The soft‐rigid hybrid design can greatly improve the handling operability of the soft large‐area HD‐sEMG electrode arrays and make it possible for mass production for widespread usage.

The electrode numbers of the 64‐channel soft HD‐sEMG electrode array are labeled in Figure S1 (Supporting Information), where electrodes 1 to 24 and 41 to 64 (the row or column inter‐distance, 11 mm) are designed to capture muscle activities in the left and right regions of the infrahyoid muscle, respectively, and electrodes 25 to 40 (the row and column inter‐distance, 5.5 and 11 mm) are used for the submental muscle. Each circular sensing electrode consists of two concentric rings (with outer diameters of 5.4 and 3.2 mm, respectively and a width of 1 mm) and six evenly distributed rays (with a width of 0.5 mm). This grid pattern was designed to save the conductive Ag/AgCl paste and enhance the surface roughness of the electrode to form a strong bond with the conductive biogel film above, as the biogel film was filled into the cavities of the grid sensing electrodes in a liquid state at 70 °C and solidified at room temperature. The electrode size and spacing were considered to be within a reasonable range based on previous studies.^[^ ^24^, ^28^ ^]^ The sizes of the sensing area are ≈1684.51 mm^2^ for submental muscles and 5414.91 mm^2^ for infrahyoid muscles, ensuring sufficient coverage of entire swallowing muscles. To ensure consistency in electrode placement on the submental and infrahyoid muscles, the two soft sensing arrays were connected via a soft and foldable connection area to form a compact patch, as shown in Figure 2D. In addition, the soft, foldable connection area will further enhance the conformability of the electrode array to large, irregular deformable surfaces, especially when the two measured areas of the submental and infrahyoid muscles are not in the same plane and have visible relative motions. Figure 2E shows an example illustrating the advantage of the soft, foldable connection area by perfectly laminating the electrode array at right angles of a structure surface.

Figure 2F shows a cluster of the fabricated 64‐channel soft HD‐sEMG electrode array patches, which were prepared by screen printing of stretchable Ag/AgCl paste and wear‐resistant carbon paste on a soft PU film weakly bonded to a stiff PET supporting layer, followed by the sequential lamination of the double‐side adhesive layer and the release PET protective layer. The flexible interconnects and the stiff contact pads were achieved by sequentially bonding PET films of various thickness to provide a simple and reliable electrical interface to the external data acquisition setup for signal transmission. The carbon paste was adopted here to enhance the scratching resistance property of the contact pads. The detailed fabrication process is schematically illustrated in Figure S2 (Supporting Information) and is also described in Experimental Section. Figure 2G shows the direct connection of the soft large‐area HD‐sEMG electrode array with a commercial cable of the HD‐sEMG acquisition setup through a home‐made FPC adaptor.

Benefiting from the soft, thin mechanical properties, strong electrode‐skin adhesive (Figure 2H) and the design of the soft, foldable connection and the temporary PET supporting/protective layers, the soft HD‐sEMG electrode array can be effortlessly attached to the large, irregular areas of the submental and infrahyoid muscles. As shown in the snapshots in Figure S3 (Supporting Information), the entire integration process mainly consists of four simple steps: i) releasing the PET protective film to exposure the double‐side adhesive, ii) placing the two sensing arrays at the targeted position of the submental and infrahyoid muscles and then gently pressing them to tightly adhere to the skin, iii) peeling off the PET temporary supporting film, and iv) connecting the HD‐sEMG electrode array to the data acquisition module via the FPC adapter. Figure 2I shows a photograph of the soft HD‐sEMG electrode array that adhered perfectly to the complex epidermal surface of the submental and infrahyoid muscles, even when the electrode array was subjected to substantial skin deformations, such as stretching (looking up) and compression (looking down) (Figure 2J). To simply evaluate the biocompatibility for long‐term wearing, the neck skin of a healthy subject was observed after wearing the soft HD‐sEMG electrode array for 24 h. It is shown that the skin can completely recover to its initial state after 1 h (Figure 2K), indicating that the electrode patch will not cause significant irritation to human skin.

### 2.2 Mechanical and Electrical Characteristics of the Soft, Large‐Area HD‐sEMG Electrode Array

**Figure 3:**
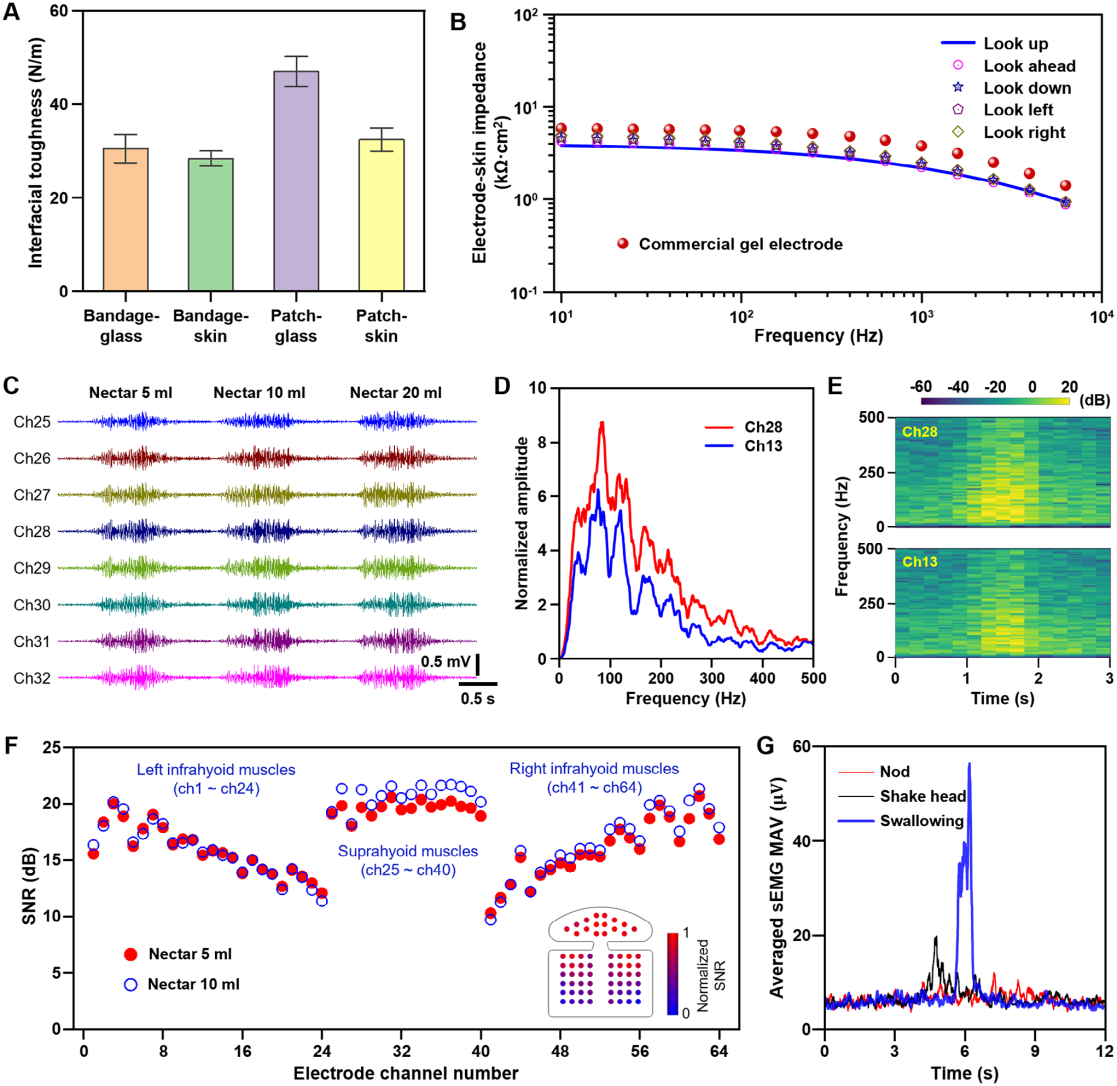
Mechanical and electrical characteristics of the soft, large‐area HD‐sEMG electrode array. A) The measured interfacial toughness of the electrode array and commercial skin bandage. B) The electrode‐skin interface impedance under various given frequency. C) Representative sEMG signals collected from a subject during swallowing of 5, 10, and 20 ml of Nectar. D,E) The calculated spectrum characteristics of collected sEMG signals from two adjacent electrodes (channels 28 and 13). F) SNR distribution of 64 channels calculated from the collected HD‐sEMG signals while swallowing 5 and 10 ml Nectar. G) The MAV of sEMG signals from a participant during three representative activities of nodding, shaking, and saliva swallowing.

The adhesion strength and mechanical stretchability are two key properties to maintain intimate contact of the electrode array to the skin. To quantitatively examine the adhesion strength, the 90‐degree peeling tests were first carried out by a universal material testing machine (INSTRON) at a speed of 6 mm min^−1^. As a comparison, a medical skin bandage (3M Tegaderm film) was selected. The electrode array and the medical skin bandage were cut into specimens of 1 cm × 10 cm, and then bonded to the cotton‐cleaned glass slide and a fresh pig skin for adhesion tests. **Figure** **3**A shows the measured interfacial toughness, which was calculated by averaging the experimental results of the measured adhesion force (Figure S4, Supporting Information). It is shown the interfacial toughness of the electrode patch with the glass slide (46.93 ± 3.24 N m^−1^) and the pig skin (32.41 ± 2.50 N m^−1^) is strong and as large as that of the commercial skin bandage. Figure S5 (Supporting Information) shows photographs of soft PU/double‐side adhesive composite film of the electrode array under various uniaxial tensile strains. The results show that the composite film exhibits excellent stretchability with an elastic strain of 30% and a fractures strain of 600%. The stress‐strain curve in Figure S6 (Supporting Information) gives the elastic modulus of ≈3.85 MPa, which is much smaller than that of the PI film (≈2.4 GPa), commonly used in previous flexible HD‐sEMG electrode array.^[^ ^28^, ^30^ ^]^

In addition, the main challenges for large‐area HD‐sEMG electrode arrays adhered on the human neck are the complex surface shape of the skin and the substantial skin deformation caused by a wide range of neck movements. These factors may result in poor electrode‐skin contact and unexpected motion artifacts. To evaluate the ability of the 64‐channel soft large‐area HD‐sEMG electrode array to measure sEMG signals over a large area under dynamic deformations, the electrode‐skin interface impedances, a key factor affecting the quality of the acquired sEMG signals, were measured over the frequency range of 10 Hz to 10 kHz under five head movements (Figure S7, Supporting Information), as shown in Figure 3B. The results show that under various neck movements, the interface impedance of the soft HD‐sEMG electrode array only increased slightly compared to the original state (i.e., looking up posture), and was still comparable to that of commonly used commercial gel electrodes, demonstrating the reliability of the interface even under large skin deformations.

To further check the ability of the electrode array to capture muscle activity during swallowing, HD‐sEMG signals were collected from a subject during swallowing of 5, 10, and 20 ml of Nectar, respectively (Figure S8, Supporting Information). The sEMG signal was measured at 1000 Hz sampling frequency and filtered by a fourth‐order Butterworth filter with a bandwidth of 20 to 500 Hz and a notch filter centered with 50 Hz and its harmonics. The representative sEMG signals of channel 25 to 32 are shown in Figure 3C. To examine the acquired signal quality, the spectrum characteristics of the two adjacent channels (Channel 28 and 13) of collected sEMG signals were analyzed, as plotted in Figure 3D,E. The results show that the shapes of the two spectrum curves are very similar, and the main frequencies are concentrated in the range of 20 to 250 Hz, consistent with the frequency distribution of sEMG signals. The signal‐to‐noise ratio (SNR) is another key parameter to assess the signal quality. Figure 3F plots the SNR in 64 channels calculated from the collected HD‐sEMG signals while swallowing 5 and 10 ml Nectar. The results show that the soft large‐area HD‐sEMG electrode array could maintain high sensitivity to detect sEMG signals with a slight swallowing. The spatial distribution characteristics of the SNR are symmetrical. The SNR values covering the submental muscles are not much different, while that adhered on the infrahyoid muscles decrease from the inside to outside, which is consistent with the physiological mechanism.

In addition, the potential interference arising from involuntary head movements such as nodding and shaking may affect signal acquisition. To study the effect of this interference, sEMG signals were collected from subjects during three representative activities: nodding, shaking, and saliva swallowing, and the corresponding mean absolute value (MAV) was compared, as shown in Figure 3G. It is found that the MAV of the nodding action is basically the same as that of the noisy signal. The MAV of the shaking head action is slightly larger over a short period, which may be caused by the exertion of neck muscles when changing direction. The MAV of the saliva swallowing motion is the largest, almost three times higher than that of the shaking head action. These results indicate that the 64‐channel soft large‐area HD‐sEMG electrode array is less susceptible to unconscious head movements, which ensures the reliable collection of high‐fidelity sEMG signals for diagnosing patients with dysphagia under various clinical settings.

### 2.3 Comparisons of HD‐sEMG Signals Collected from Different Dysphagia Patients

To verify the feasibility of the soft large‐area HD‐sEMG electrode array for dysphagia assessment, HD‐sEMG signals were first acquired in nine swallowing tasks from 38 participants recruited from the Department of Rehabilitation, the First Affiliated Hospital School of Medicine, Zhejiang University. The severity of these participants was divided into four levels, including the none (represented by 12 healthy young adults), the mild (represented by 14 healthy elderly adults with a natural aging in swallowing muscle function), the moderate or severe groups (represented by 12 clinically diagnosed post‐stroke dysphagia patients), as summarized in Table S1 (Supporting Information). Nine swallowing tasks were adopted based on the volume‐viscosity swallow test,^[^ ^35^ ^]^ corresponding to various combinations of swallowing food of three different viscosities (Nectar, Liquid, and Pudding) and three different volumes (5, 10, and 20 ml). The Nectar (or Pudding) was made by mixing edible compound thickener (SoftiaS) of 3 grams and purified water of 120 ml (or 60 ml), and the Liquid was made of purified water. For the healthy young participants in the none group and the healthy elderly participants in the moderate group, HD‐sEMG signals were collected under all nine different swallowing tasks, and each subject performed each task at least three times. For patients with dysphagia, they were first assessed into the moderate dysphagia or severe dysphagia group according to whether they coughed when swallowing 5 ml Liquid. HD‐sEMG signals were then collected under seven swallowing tasks (except for 10 ml and 20 ml Liquid) for the severe dysphagia patients and under eight (patients coughed when swallowing 10 ml Liquid, skipping 20 ml Liquid) or nine swallowing tasks for the moderate dysphagia patients. Each subject performed each task at least 2 times. The detailed swallowing tasks for the four groups of participants are summarized in Table S2 (Supporting Information).

**Figure 4:**
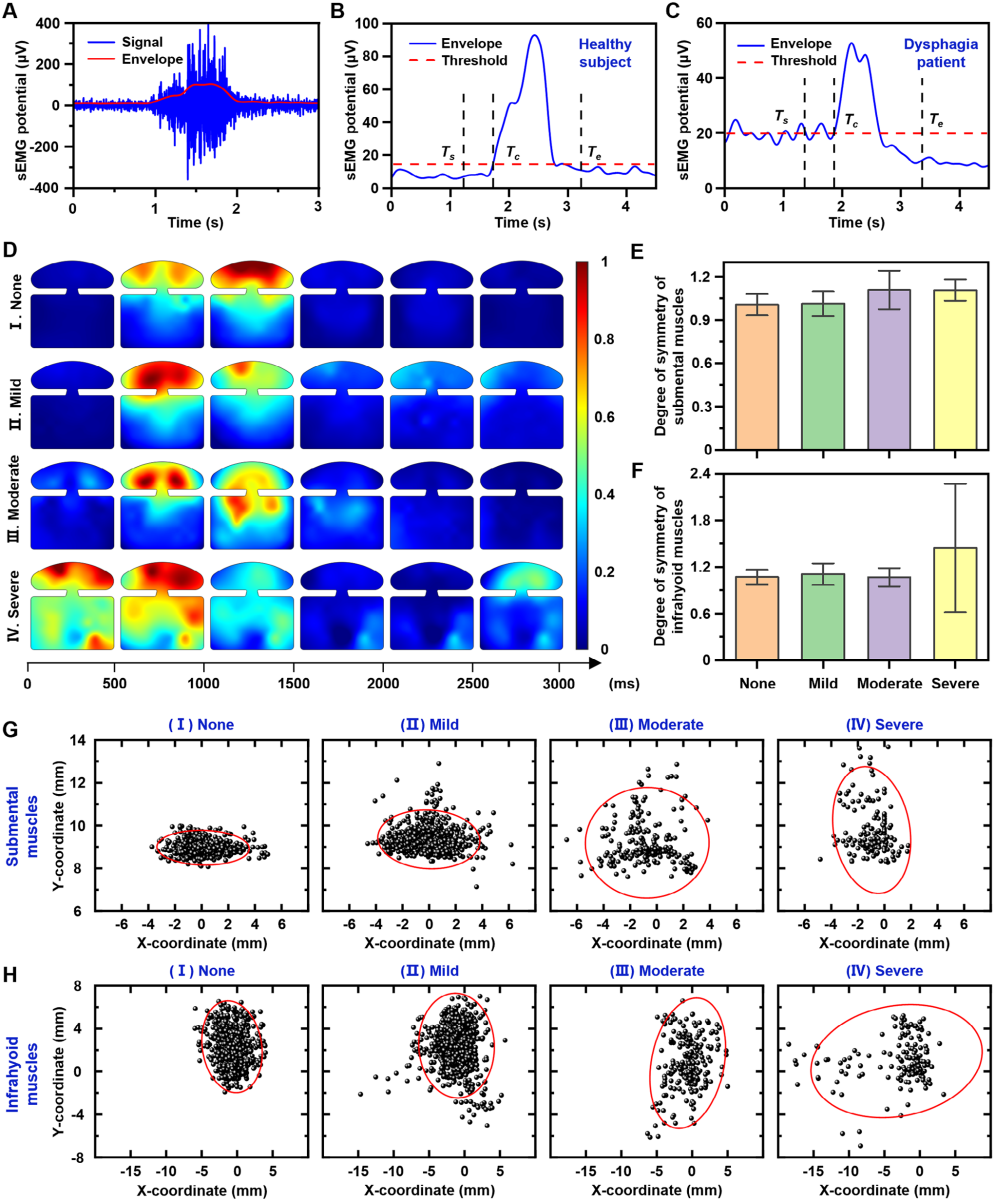
Comparison of HD‐sEMG signals collected from different dysphagia patients. A) A normal sEMG signal and its envelope curve. Representative envelope curve of sEMG signals during swallowing for B) the healthy participants and C) the dysphagia participants. D) Representative muscle activity intensity distribution maps of participants with different dysphagia severity during the swallowing of 10 ml Nectar. The calculated degree of symmetry indicators for E) the submental muscles and F) the infrahyoid muscles of participants with different dysphagia severity. Scatter plots and 95% confidence ellipses of the muscle activity centroids of G) the submental muscles and H) the infrahyoid muscles with different dysphagia severity.

To identify the muscle activation time and intensity for automatic extraction of 64‐channel HD‐sEMG signals in various swallowing tasks, the envelope curve of sEMG signals during swallowing was calculated to capture the changing trend of the signal amplitude (**Figure** **4**A), and two representative swallowing modes were found, corresponding to the healthy and dysphagia participants, respectively. In healthy participants, facial muscles were primarily used to hold the liquid bolus during the oral preparation phase. Thus, the signal amplitude on both sides of the envelope is low and flat because the hyoid muscles are not activated (Figure 4B). In this case, the mean value of the envelope of the 1500‐ms resting phase plus 3 times its standard deviation was set as a threshold. In contrast, for participants with dysphagia, additional assistance from the hyoid muscles was required during the initial oral preparation phase. Therefore, the left side of the envelope reflects the contraction signal of the hyoid muscle, and the right side represents the resting signal (Figure 4C), and the mean value of the envelope of the 1500‐ms contraction phase plus 3 times its standard deviation was set as a threshold. When the amplitude reaches the threshold, the swallowing task is considered to have started, labeled as *T_c_*. The corresponding start time *T_s_* and end time *T_e_* of swallowing are then defined as *T_s_ = T_c_* – 500 and *T_e_ = T_c_* + 1500, respectively, for automatic capture of HD‐sEMG signals during a series of swallowing tasks. This greatly improves data processing efficiency, and reduces the influence of human errors and subjective factors and ensures the reliability of the subsequential analysis.

By analyzing and comparing the collected HD‐sEMG signals, several quantitative indicators for dysphagia assessment were obtained. Figure 4D presents the representative muscle activity intensity distribution maps of people with different dysphagia severity during the task of swallowing 10 ml Nectar. It is shown that for the healthy young subjects, the muscle activation started in the second frame and then spread from the submental muscle to the infrahyoid muscle until the maximum contraction was reached in the third frame, at which time the submental and infrahyoid muscles showed an almost symmetrical distribution of muscle activity. Then, all muscles immediately returned to their original resting state. These indicated that healthy young subjects can rapidly complete swallowing without the assistance of the hyoid muscles when holding the food bolus in the oral preparation stage, and the muscle activity is highly symmetrical. For the mild subjects, the muscles were not activated in the first frame, but immediately contracted to the maximum in the second frame, with the center of activity located at the lower right of the submental muscle (the left side of the figure corresponds to the right side of the subject). The overall muscle activity intensity decreased in the third frame and then gradually returned to the initial resting state. The swallowing activity in mild subjects was similar to that of healthy young subjects, but slightly weaker. Unlike the normal cases, the sEMG signal in the mild case does not completely return to the resting state even after 2500 ms. This may be attributed to a compensatory mechanism, where certain muscle groups maintain a higher level of activation for an extended period to stabilize the swallowing process. This prolonged activation might reflect a subtle neuromuscular deficit or a delay in the muscle relaxation phase.

For the moderate subjects, the muscles exhibited a low‐intensity activity state in the first frame and the submental muscles immediately contracted to the maximum in the second frame. In the third frame, the activity intensity of the submental muscles decreased while that of the infrahyoid muscles increased significantly. In the fourth frame, swallowing was still not completed, and the infrahyoid muscles were still needed to continue to transport the food bolus. subsequently, all muscles basically returned to the initial resting state. For the severe subjects, the muscles were highly activated in the first frame and the entire hyoid muscles continued to swallow heavily in the second frame. In the third frame, the intensity of muscle activity began to decrease but the infrahyoid muscles remained in a low‐intensity activity state. In the last frame, the submental muscles began to contract strongly again for starting the second swallowing. This reflects that the severe dysphagia subjects cannot hold the food bolus with facial muscles alone, and require strong assistance from the hyoid muscles. The swallowing requires simultaneous rather than sequential mobilization of the submental and infrahyoid muscles, and needs to be completed in multiple swallowing. The muscle activity on the left and right sides is extremely asymmetric during the whole process.

As visually revealed by the muscle activity characteristics in Figure 4D, the symmetry of the human body's physiological structure determines the symmetry of muscle activity during swallowing. For patients with dysphagia, due to anatomical abnormalities or neurological dysfunction, they tend to swallow unilaterally, resulting in an asymmetric distribution of muscle activity. To quantitatively describe the symmetry of bilateral muscle activity during swallowing, the degree of symmetry (*DOS*) indicator was calculated, as described in Experimental Section. The closer the *DOS* indicator is to 1, the more balanced the muscle activity on the left and right sides of the subject is, and the more normal the swallowing function is. Figure 4E,F shows the calculated *DOS* indicators for the submental and infrahyoid muscles, respectively. For the submental muscles, the *DOS* indicator of the subject with none and mild dysphagia was not much different, both close to 1. However, the *DOS* indicator of the subjects with moderate and severe dysphagia deviated from the healthy level and was close to 1.1. For the infrahyoid muscles, the *DOS* indicator ranged from 1.05 to 1.15 for subjects with no, mild, and moderate dysphagia, but the *DOS* indicator for subjects with severe dysphagia deviated greatly from the healthy level, exceeding 1.4. Therefore, the *DOS* indicator can quantitatively reflect the changes in the patient's swallowing function.

In addition, the symmetry of muscle activity during swallowing can be further described by the barycenter indicators *B_x_* and *B_y_*, which represent the barycenter of the HD‐sEMG signals to the *x* and *y* axes in the coordinate system shown in Figure S9 (Supporting Information). Figure S10 (Supporting Information) plotted the probability density distribution of the barycenter. It is showed that for the healthy young subjects, the barycenter *B_x_* and *B_y_* of muscle activity during swallowing obeys a normal distribution for the submental and infrahyoid muscles. As the dysphagia becomes more severe, its dispersion increases. By taking both the barycenter *B_x_* and *B_y_* into account in all time windows, a 2D scatter diagram was obtained for each group. To further analyze the distribution characteristics of the scatters, the confidence ellipse containing 95% data points in the plane was calculated (Figure 4G,H). The center position of the confidence ellipse was determined by the mean vector of the scatters in the *x* and *y* directions. The shape and direction of the ellipse were obtained by solving the eigenvalue and the eigenvector of the covariance matrix, respectively. The lengths of the major and minor axes of the ellipse are adjusted according to the critical value of the chi‐square distribution to meet the specified confidence level. For the healthy and mild subjects, the scatters are highly concentrated in the *x* direction for the submental muscles and in the *y* direction for the infrahyoid muscles. In contrast, for the moderate and severe subjects, the scatters are sharply dispersed. As the severity of dysphagia increases, the center shifts greatly.

### 2.4 Accurate and Noninvasive Dysphagia Assessment Assisted with Machine Learning Model

**Figure 5:**
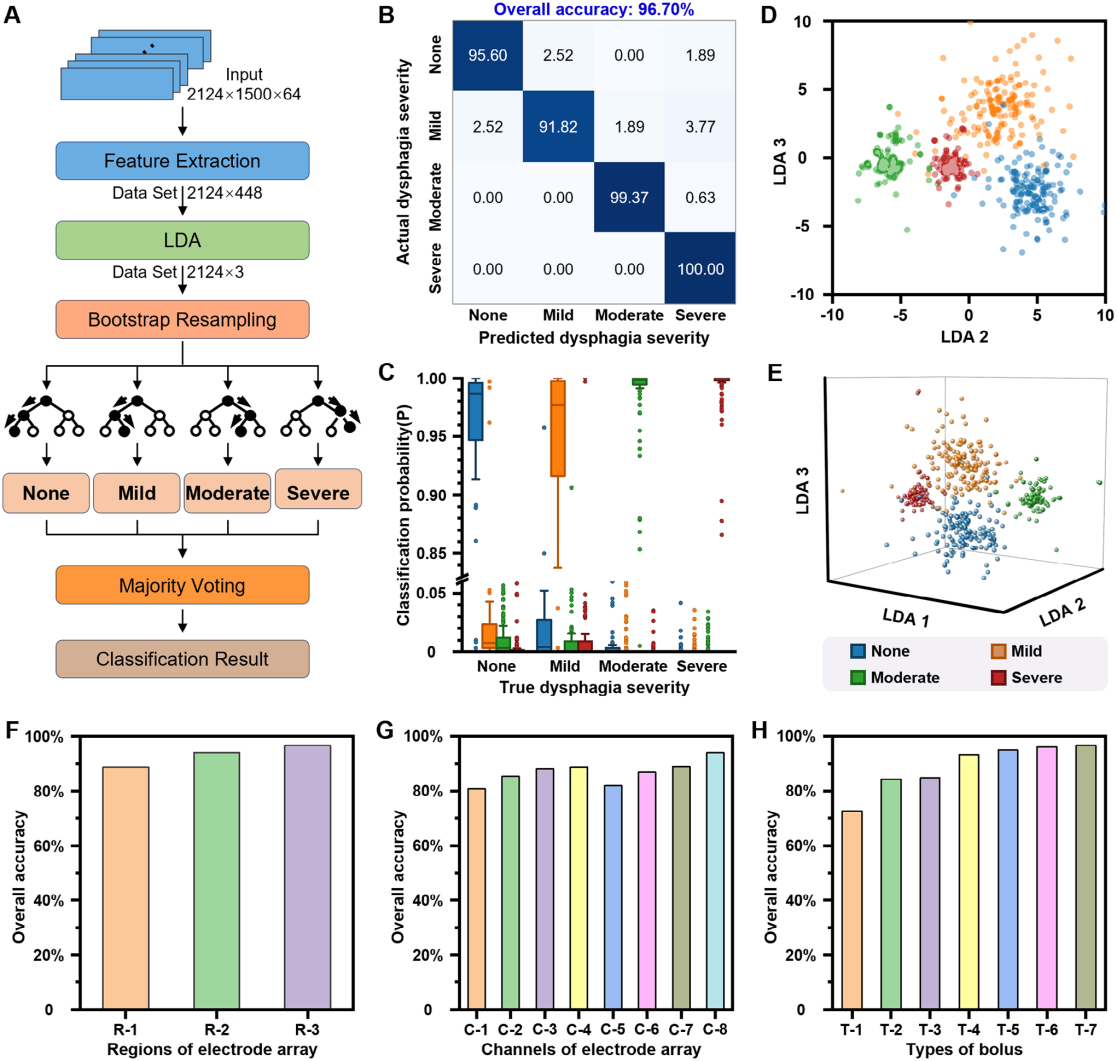
Accurate and noninvasive dysphagia assessment assisted with machine learning model. A) The overall architecture of the quantitative dysphagia assessment model. B) Confusion matrix of the recognition accuracy for the four severity levels of dysphagia. C) Sample classification probability distribution of the machine learning model. D) 2D and E) 3D scatter plots of signal features after dimensionality reduction by the LDA. Overall recognition accuracy of the model by using electrode array F) covering various muscle regions and G) with various distribution densities. H) Overall recognition accuracy of the model by using HD‐sEMG signals under swallowing different food bolus types.

Both the *DOS* and barycenter indicators were shown to effectively describe the discrepancy in muscle activity of different dysphagia patients during swallowing, demonstrating the feasibility of quantitative, objective dysphagia assessment using HD‐sEMG signals. To achieve automatic diagnosis and classification of different dysphagia severity levels, a dysphagia assessment model based on machine learning was developed by combining the linear discriminant analysis (LDA) and the random forest algorithms. The LDA algorithm was used to reduce the dimensionality of the extracted sEMG signal features and the random forest algorithm was used for subsequent classification to enhance the robustness of the model. **Figure** **5**A presents the overall architecture of our quantitative dysphagia assessment model. The detailed description of the construction of the machine learning model is presented in Experimental Section.

The final classification results of the model are shown in the confusion matrix in Figure 5B, showing an overall recognition accuracy of 96.7%. Except for the mild group, the remaining three groups are more than 95% accuracy, while the moderate and severe categories are as high as 99.37% and 100% accuracy. Each sample of the test set has model output probabilities classified as none, mild, moderate and severe. Statistically, the classification probability distribution of the severity classification model of dysphagia can be obtained, as displayed in Figure 5C. As can be seen from the figure, most samples in the four types have the maximum probability of being classified as themselves, and their median probability is more than 0.97, which is consistent with the classification results of more than 90% recognition accuracy of the four levels in the confusion matrix. Besides, in terms of the probability distribution boxplot classified as its own, the box of the severe group is the shortest and highest, and the moderate group is slightly longer and shorter. The none group is longer and shorter, and the mild group is the longest and shortest. Hence the recognition accuracy of the four types in the confusion matrix is reduced in the order of Severe, Moderate, None and Mild. Figure 5D,E uses LDA for dimensionality reduction and visualizes the sEMG clustering of the swallowing process in patients with different swallowing disorders severities. In this 2D or 3D space, different clusters appear, with colors and markers distinguishing the swallowing disorder severities, indicating minimal overlap in the categories. This clear separation demonstrates the efficacy of the model in distinguishing features of various dysphagia severity. Figure S11 (Supporting Information) illustrates the performance of the dysphagia severity classification model in the receiver operating characteristic (ROC) curve and the precision rate‐recall rate (PRC) curve. The ideal ROC and PRC curves should be as close as possible to the upper left corner and upper right corner respectively, and the area under the curve is close to 1. Obviously, the results of this model are similar to the ideal situation.

Furthermore, several critical factors affecting the recognition accuracy of the model were discussed in depth, including the electrode distribution and density, and the type of food pellets. Figure S12 (Supporting Information) schematically shows possible electrode distribution and density of the electrode array, and the classification results for each case are shown in the confusion matrix in Figure S13 (Supporting Information). It is shown that the overall recognition accuracy of the model is the highest when the electrode array covers the whole hyoid muscles (Figure 5F), and increasing the number of electrode channels on the same muscles can improve the model recognition accuracy (Figure 5G). Besides, the type of food pellets is also an important objective factor affecting the model identification accuracy. We selected seven pellets shown in Table S3 (Supporting Information). The overall recognition accuracy of the seven kinds of food pellets increased successively, and the recognition accuracy of the food pellets containing multiple solutions was obviously higher than that of a single solution (Figure 5H and S13, supporting Information). In addition, for the pellets containing same number of solutions, the identification accuracy of those that contain liquid is lower.

**Figure 6:**
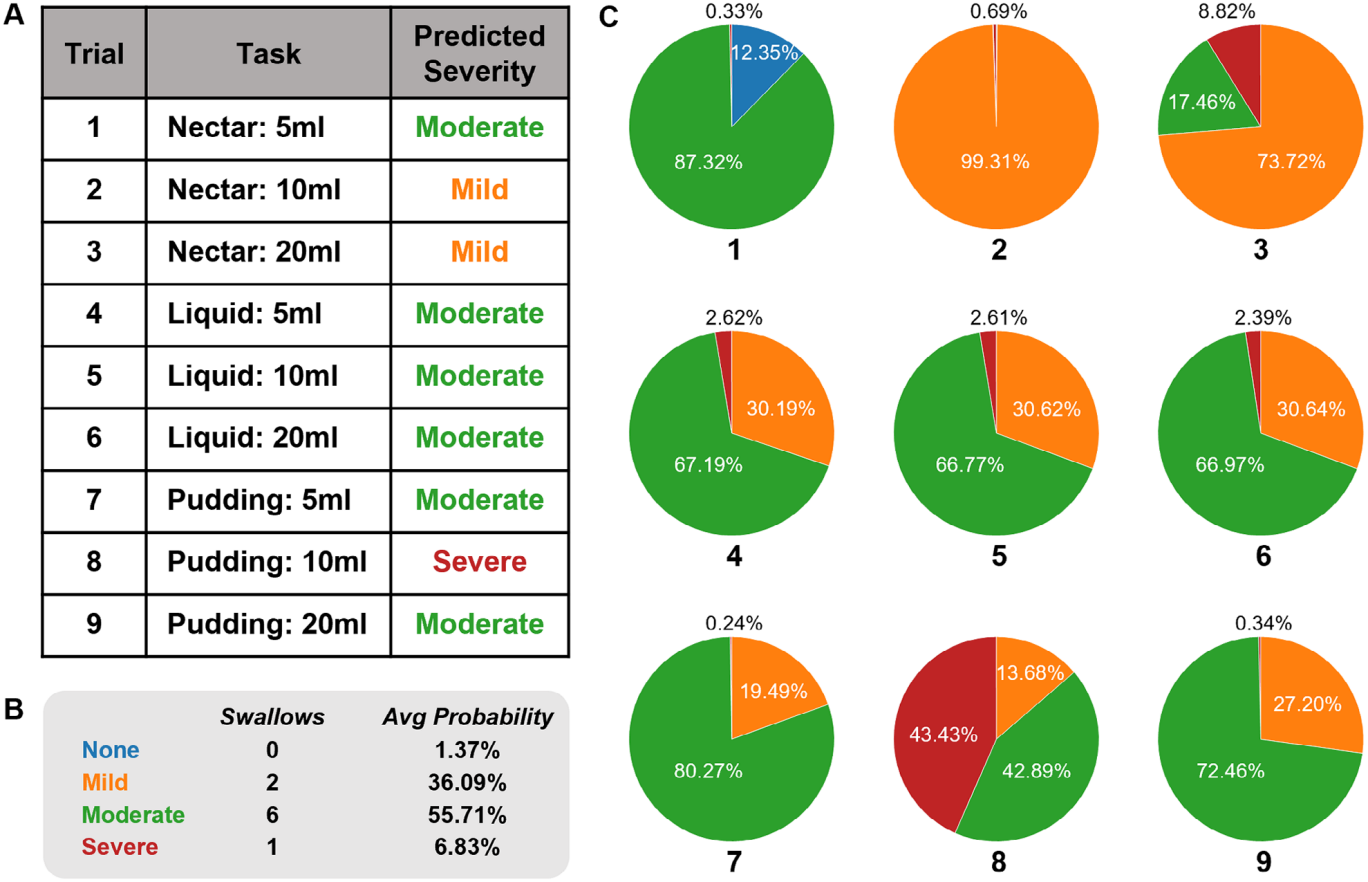
Performance test of the machine learning model on new patients. A) Comparison of the predicted results from the machine learning model and the clinically diagnosed method via V‐VST test in 9 cases. B) The probability distribution across different dysphagia classifications over the nine tests. C) A pie chart that visualizes the probability distribution across the tests.

To evaluate the practical applicability of our model, we conducted a trial on a new patient, performing a total of nine V‐VST assessments. The sEMG signals collected during each test were processed through our model. As shown in **Figure** **6**A, the model evaluation results were consistent with the artificial identification results in 7 out of 9 cases, indicating that the severity classification model of dysphagia can achieve the correct assessment on new patients and produced accurate diagnostic results. Figure 6B illustrates the probability distribution across different dysphagia classifications over the nine tests, with average probabilities recorded as follows: 1.37%, 36.09%, 55.71%, and 6.83%. Figure 6C presents a pie chart that visualizes the probability distribution across the tests, highlighting that the majority of the sample predictions aligned correctly with the patient's diagnosed dysphagia grade. These findings not only validate the robustness and accuracy of the proposed model in detecting dysphagia severity, but also underscore its potential for broader applicability in real‐world clinical and practical scenarios.

## 3 Discussion

This work has reported a soft large‐area HD‐sEMG electrode array integrated with a machine learning based classification model to accurately and automatically assess the severity of dysphagia. The soft large‐area HD‐sEMG electrode array combines tissue‐soft mechanical properties, strong skin adhesion, and easy operation, which can be seamlessly integrated with irregular, complex epidermal surface of the submental and infrahyoid muscles to fully capture muscle activities during swallowing, even when the skin undergoes substantial deformation. By encapsulating the soft, thin, self‐adhesive sensing regions with the temporary stiff PET supporting and protective layers to achieve sufficient rigidity enhancement for easy handling, the electrode arrays can be rapidly fabricated using a scalable, low‐cost screen‐printing technology. In addition, the electrode arrays feature a simple and reliable electrical interface to the external data acquisition setup for signal transmission. By using HD‐sEMG signals collected from a large number of participants, a machine learning model was trained and showed an overall recognition accuracy as high as 96.7% for automatic assessment of the severity of dysphagia. The results of this work demonstrated the effectiveness of a soft large‐area HD‐sEMG electrode array conformal to the submental and infrahyoid muscles assisted with a machine learning in the accurate, noninvasive, rapid and objective assessment of the severity of dysphagia.

Although our model performed well on the test set, its validation using only one patient limits our ability to fully assess its robustness and clinical applicability. Moreover, the variability in muscle physiological structure (e.g., muscle distribution, subcutaneous fat thickness) among diverse populations (e.g., the elderly and young adults), can affect the acquisition of sEMG signals. Addressing these factors is crucial for enhancing the robustness and reliability of the electrode arrays, ultimately improving the precision and applicability of assessments of dysphagia severity across different patient groups. Future efforts could focus on collecting sEMG signals from a larger, more diverse group of patients to further enhance the practical reliability and applicability of the model.

## 4 Experimental Section

### Design and Fabrication of the Soft Large‐Area HD‐sEMG Electrode Array

The design of the sensing area patterns for the HD‐sEMG electrode array was primarily based on the anatomical structures of the submental and infrahyoid muscles, which were the main muscle groups involved in swallowing disorders. Specifically, the submental muscles resemble an inverted triangle, while the infrahyoid muscles resemble a rectangle. Based on these observations, the two sensing area patterns were designed and incorporated a soft, foldable connection structure to connect the two areas. This design decouples the coupled deformation caused by the device's attachment to the skin surface, ensuring better conformity and adhesion to the complex target regions.

The fabrication of the soft large‐area HD‐sEMG electrode array began with the preparation of a soft PU film that was weakly bonded to a stiff PET substrate. Stretchable Ag/AgCl paste and carbon paste were then patterned on the soft PU film by screen printing technology to form stretchable traces and wear‐resistant contact pads, and baked on a hot plate at 135 °C for 30 min. The soft on‐skin electrode array and flexible interconnects were encapsulated by sequential bonding of a double‐sided adhesive layer/ PET protective layer composite and a thin PET film, respectively. Then, a thick PET film was tightly bonded to the back of contact pads via a double‐side adhesive (DP25‐50TA, Zhongshan New Asia Adhesive Products) to provide a simple and reliable electrical interface to the external data acquisition setup for signal transmission. Finally, the soft large‐area HD‐sEMG electrode arrays were cut by a laser cutting machine to define the layout.

### Integration of the Tough, Stretchable Conductive Biogel with the Electrode Array

Gelatin (Aladdin) was added into deionized water at a weight ratio of 15 wt.% and soaked for 1 h. Then, sodium pyrrolidone carboxylate (PCA‐Na, Macklin) was added to the above solution (weight ratio of PCA‐Na, 40 wt.%), and the mixture was stirred for 1 h at constant temperature of 50 °C to obtain a homogeneous precursor. Finally, black food coloring was added to the precursor and stirred to obtain the tough, stretchable phase‐change conductive biogel. The solidified conductive biogel was subsequently heated in 70 °C water to obtain precursors and filled into the cavities of the grid sensing electrodes of the electrode array (Figure S15, Supporting Information). When cooled to room temperature, the conductive biogel precursor solidified again to form conductive films and were tightly integrated to the sensing electrodes.

### Standardized Electrode Placement Protocols

Differences in laryngeal anatomy, such as the size and prominence of the laryngeal prominence, could influence the conformability and placement of the electrode array. To accurately control the placement of the electrode array, standardized electrode placement protocols were implemented using clearly defined anatomical landmarks, guided by experienced clinicians. First, the subjects were instructed to tilt their heads back until they felt a noticeable tightening of the skin. Then, the hyoid bone was located using ultrasound, and the position was marked on the skin. Finally, the electrode array was vertically placed in the corresponding marked area, ensuring that the two measurement regions of the HD‐sEMG electrode array correspond to the submental and infrahyoid muscle groups, respectively.

### Procedure of Volume‐Viscosity Swallow Test

The volume‐viscosity swallow test (V‐VST) evaluates how increasing viscosity impacts patients' swallowing efficacy and safety. The test begins with nectar viscosity, progressing through boluses of 5, 10, and 20 ml, each incrementally increasing in difficulty. If the patient completes the nectar series without significant symptoms of aspiration (such as coughing or a drop in oxygen saturation ≥3%), a more challenging liquid viscosity series was tested, with boluses of 5, 10, and 20 ml. Following this, a safer pudding viscosity series was assessed in the same manner.

If a patient exhibits signs of impaired safety during the nectar viscosity series, the test was paused, and the liquid viscosity series was skipped. Instead, the pudding viscosity series was evaluated. If the pudding viscosity was found to be safe and no residue was detected, it was recommended as the preferred option. If the patient demonstrates issues with the liquid viscosity series, the liquid series was halted, and the pudding viscosity series was tested. In this scenario, the optimal nectar viscosity volume was recommended for the patient's safety.

### Calculations of the Three Quantitative Indicators for Dysphagia Assessment

The muscle activity intensity distribution map clearly displays the spatiotemporal distribution information of muscle activity, which was important for identifying swallowing abnormalities and providing insightful analysis for quantitative dysphagia assessment. To obtain the distribution maps of muscle activity intensity in people with different dysphagia severity, 3000 ms of HD‐sEMG signals were automatically captured from the moment *T_s_*, when the subject performed a specific swallowing task, and then equally divided into 6 segments. The RMS values of 64 channels in each segment were calculated and normalized. Then, the biharmonic spline interpolation method was used to spatially expand the normalized RMS value in a single time window to obtain continuous distribution of the normalized RMS value. Finally, red and blue were used to represent the maximum and minimum RMS values, and six consecutive frames of muscle activity intensity distribution were plotted.

To calculate the degree of symmetry indicator, 1500 ms of HD‐sEMG signals were automatically captured from the moment *T_c_*, and then equally divided into 15 segments. The RMS values of 64 channels in each segment were calculated. The 32 channels on the left sides and on the right side were matched according to the symmetry relationship, and the RMS ratios were calculated in sequence. Finally, the 15 × 8 RMS ratios of the submental muscles and the 15 × 24 RMS ratios of the infrahyoid muscles were averaged to obtain the symmetry of the muscle activity in the two areas during the subjects' swallowing process. The degree of symmetry (*DOS*) indicator was calculated as

(1) $$DOS=\frac{1}{N\times M}\sum \frac{RMS_{\mathrm{left}}}{RMS_{\mathrm{right}}}$$

where *RMS*_left_ and *RMS*_right_ are the RMS value of the left and right channels with a geometrically symmetrical relationship, and *N* and *M* are the number of the segments and the RMS ratio.

The symmetry of muscle activity during swallowing could be further described by the barycenter indicator of the HD‐sEMG signals, which could be calculated as

(2) $$B_{x}=\frac{\sum_{i=1}^{n}RMS_{i}\cdot x_{i}}{\sum_{i=1}^{n}RMS_{i}},\;B_{y}=\frac{\sum_{i=1}^{n}RMS_{i}\cdot y_{i}}{\sum_{i=1}^{n}RMS_{i}}$$

where *x_i_* and *y_i_* represent the horizontal and vertical coordinates of a channel in the coordinate system. *B_x_* and *B_y_* are the barycenter to the *x* and *y* axes. *RMS_i_* is the RMS value of a channel. *n* is the channel number, which equals 16 for the submental muscles and 48 for the infrahyoid muscles, respectively.

### Construction of the Machine Learning Based Dysphagia Assessment Model

The classification process of sEMG signals recorded during swallowing employs a machine learning approach with two main stages. First, Linear Discriminant Analysis (LDA) was utilized to reduce the dimensionality of sEMG signals for feature extraction. Second, the Random Forest (RF) algorithm was applied to classify the processed signals, enhancing the model's robustness. In this study, a V‐VST assessment was first conducted, capturing sEMG signals from healthy adults and patients with three dysphagia severity levels. To address class imbalance in the sample distribution, Gaussian white noise was added to augment the dataset, resulting in 2124 sEMG samples. Each sEMG sample underwent a series of preprocessing steps, including filtering and noise reduction, followed by feature extraction. Seven features were calculated, including kurtosis, peak width at half maximum, sample entropy, power spectra peak frequency, power spectra normalized peak frequency, power spectra total power, and power spectra centroid. These features were chosen for their physiological relevance, as they capture key aspects of muscle activation during swallowing, including measures of signal amplitude, frequency content, and temporal dynamics, which were critical for characterizing the neuromuscular activity underlying dysphagia. Moreover, some of these selected features had been demonstrated to be effective as biomarkers of dysphagia in the literature, as shown by Brien et al.^[^ ^36^ ^]^

This feature extraction process generated a dataset with 448 features, encompassing seven attributes for each of the 64‐channel sEMG. LDA was subsequently employed for dimensionality reduction, involving the computation of class mean vectors and the derivation of within‐class and between‐class scatter matrices based on these vectors. Through eigen decomposition, the leading 2/3 of eigenvectors were selected, projecting the original dataset onto these eigenvectors to achieve dimensionality reduction. This process yielded a dataset characterized by 3D features, facilitating subsequent classification analyses. The simplified 3D features dataset was split into training and test sets in a 70%–30% ratio, with random sampling employed to ensure data representativeness and variability.

RF method was employed for classification. To enhance the predictive performance of the RF model, a genetic algorithm (GA) was employed to optimize the key hyperparameters, include the number of trees (*N_estimators*), maximum depth (*Max_depth*), minimum samples for splitting a node (*Min_samples_split*), and minimum samples for a leaf node (*Min_samples_leaf*). These hyperparameters were initially set within the following ranges: *N_estimators* ranged from 10 to 500 with a step size of 10, *Max_depth* from 5 to 50 with the option of no depth limit, *Min_samples_split* from 2 to 10, and *Min_samples_leaf* from 1 to 10. The GA was applied over 40 generations with a population size of 50, and cross‐validation was used as the fitting function to evaluate the generalization performance of the model. The tuning process resulted in the following optimized hyperparameters: *N_estimators* = 500, *Max_depth* = 15, *Min_samples_split* = 2, and *Min_samples_leaf* = 2. After optimizing the hyperparameters, the model was retrained and evaluated on the test set, leading to improved accuracy and other key performance metrics, including the confusion matrix. The RF method introduces perturbation by bootstrapping sample subsets and randomly selecting a subset of features at each node to identify the optimal split criterion, thereby enhancing the classification model's robustness and accuracy.

### Strategies to Reduce the Intra‐ and Inter‐Individual Differences

Normal swallowing indeed exhibits significant variability both within individuals (intra‐individual) and across various individuals (inter‐individual), posing a challenge in accurately modeling swallowing behavior. To address this issue, a diverse group of participants was recruited to capture a wide range of normal swallowing patterns, ensuring that the model was robust against naturally occurring individual differences. Each participant performed multiple swallowing trials under standardized conditions, allowing us to capture intra‐individual variability and mitigate the effect of momentary fluctuations. Moreover, comprehensive spatial coverage was ensured using a 64‐channel electrode array, which effectively captures variations in muscle anatomy. The dense sensor array also ensure that some slight position deviations would not significantly affect the authenticity of the signals and the recognition accuracy of the model.

Additionally, normalization techniques were applied to the extracted features to reduce inter‐individual differences in signal amplitude caused by variations in subcutaneous tissue thickness or muscle morphology. Finally, the random forest model employed was inherently robust to noise and variability, and further accommodating both intra‐ and inter‐individual differences. These strategies had significantly enhanced the reliability of the analysis and reduced the intra‐ and inter‐individual differences.

### Ethics Statement

All experiments on human skin were approved by the Human Research Ethics Committee of Zhejiang University. Informed consent was obtained from all participants.

## Conflict of Interest

The authors declare no conflict of interest.

## Author Contributions

W.H., L.M., and K.L. contributed equally to this work. C.W., D.W., J.S., and Z.C. conceived the idea. W.H., L.M., K.L., C.H., and Y.S. performed experiments. W.H., K.L., S.Z., and C.W. analyzed the data. C.W., D.W., J.S., and Z.C. supervised the project. W.H., K.L., and C.W. wrote the manuscript. D.W., J.S., and Z.C. revised the manuscript. All authors took participate in the discussion.

## Supporting information



Supporting Information

## Data Availability

The data that support the findings of this study are available from the corresponding author upon reasonable request.
