# Supplementary material for: Accurate and Noninvasive Dysphagia Assessment via a Soft High‐Density sEMG Electrode Array Conformal to the Submental and Infrahyoid Muscles
Source: Adv Sci (Weinh). 2025 Mar 24;12(25):2500472. doi: 10.1002/advs.202500472 (PMC12224961; doi:10.1002/advs.202500472)
Supplement: Supplementary file 1 — Supporting Information [file ADVS-12-2500472-s001.pdf]

ADVANCED SCIENCE
Open Access

Supporting Information

for *Adv. Sci.*, DOI 10.1002/advs.202500472

Accurate and Noninvasive Dysphagia Assessment via a Soft High-Density sEMG Electrode Array Conformal to the Submental and Infrahyoid Muscles

Weijie Hong, Lin Mao, Kai Lin, Chongyuan Huang, Yanyan Su, Shun Zhang, Chengjun Wang, Daming Wang*, Jizhou Song* and Zuobing Chen**

Supplementary Materials for

Accurate and Noninvasive Dysphagia Assessment via a Soft High-Density sEMG Electrode Array Conformal to the Submental and Infrahyoid Muscles

Weijie Hong[1,2,3#], Lin Mao[1#], Kai Lin[2#], Chongyuan Huang[2], Yanyan Su[2], Shun Zhang[1,2], Chengjun Wang[1,2,3*], Daming Wang[1*], Jizhou Song[1,2,3*], Zuobin Chen[1*]

[1]*Department of Rehabilitation Medicine, The First Affiliated Hospital School of Medicine, Zhejiang University, Hangzhou, 310003, China*

[2]*Key Laboratory of Soft Machines and Smart Devices of Zhejiang Province, State Key Laboratory of Brain-Machine Intelligence, Department of Engineering Mechanics, Zhejiang University, Hangzhou 310027, China*

[3]*Huanjiang Laboratory, Zhuji 311899, China*

[#] These authors contributed equally to this work.

Email: wangchj@zju.edu.cn (C. Wang), 1719001@zju.edu.cn (D. Wang), jzsong@zju.edu.cn (J. Song) and czb1971@zju.edu.cn (Z. Chen).

This PDF file includes:

- Supplementary Figures S1 to S15
- Supplementary Tables S1 to S3

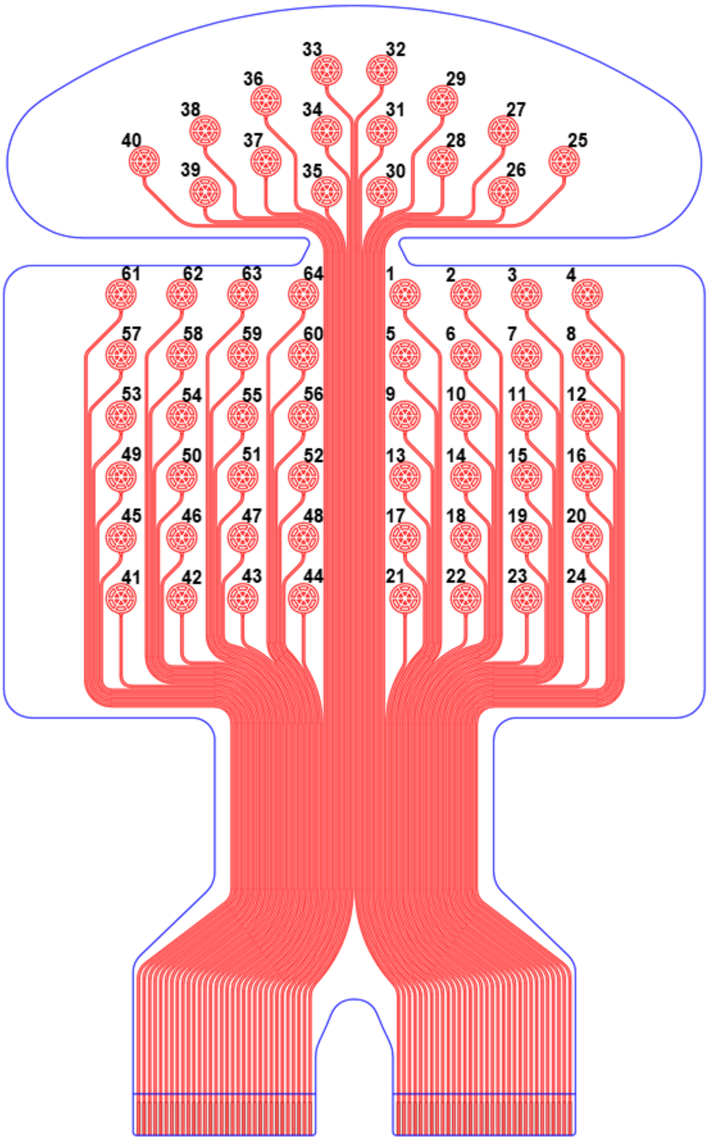

Figure S1. The schematic layout and the electrode number of the 64-channel soft large-area HD-sEMG electrode array.

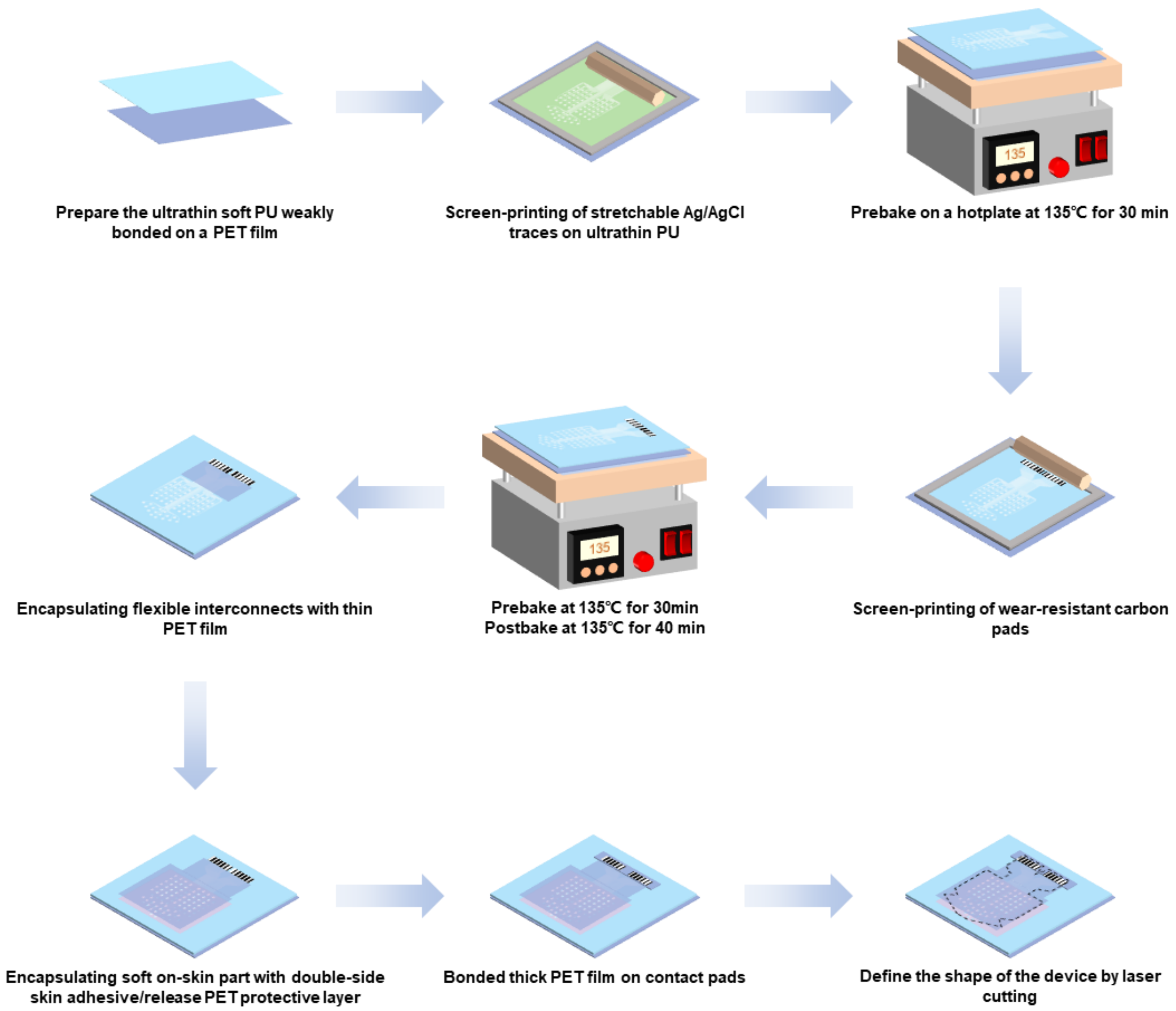

Figure S2. The schematic flow chart of the layer-by-layer fabrication process of the soft large-area HD-sEMG electrode array.

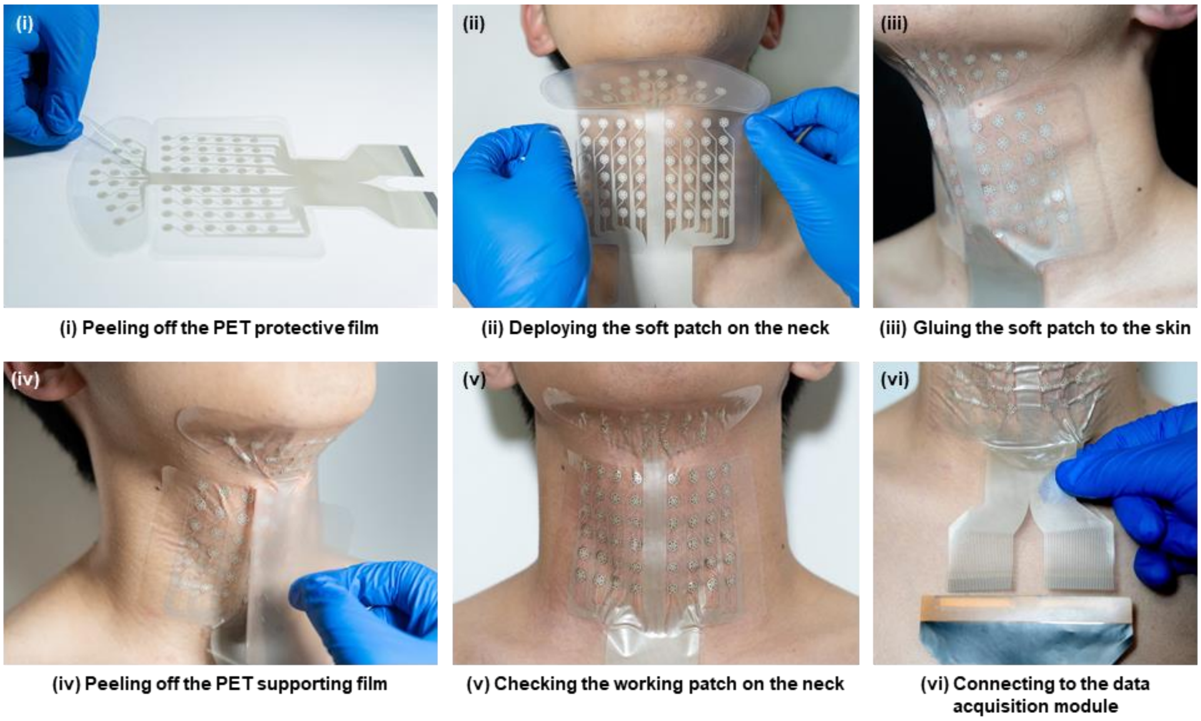

Figure S3. The snapshots showing the simple and convenient integration process of the soft large-area HD-sEMG electrode array with the large, irregular areas of the submental and infrahyoid muscles.

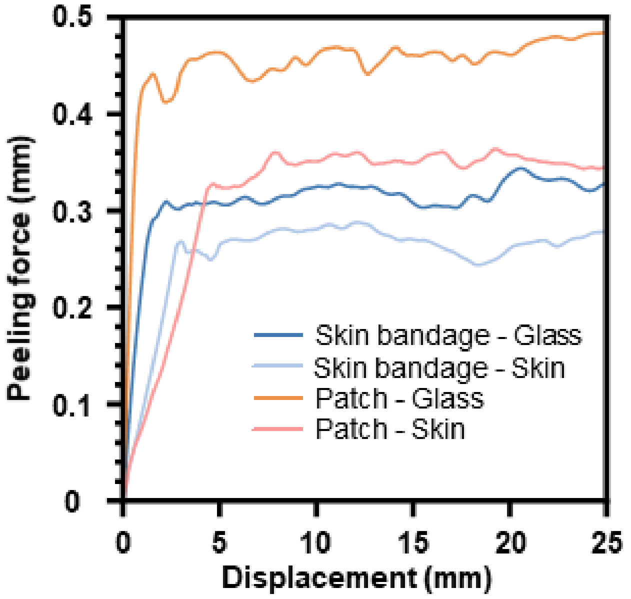

Figure S4. The representative measured adhesion forces of the electrode array (or the skin bandage) with the skin and the cleaned glass slide.

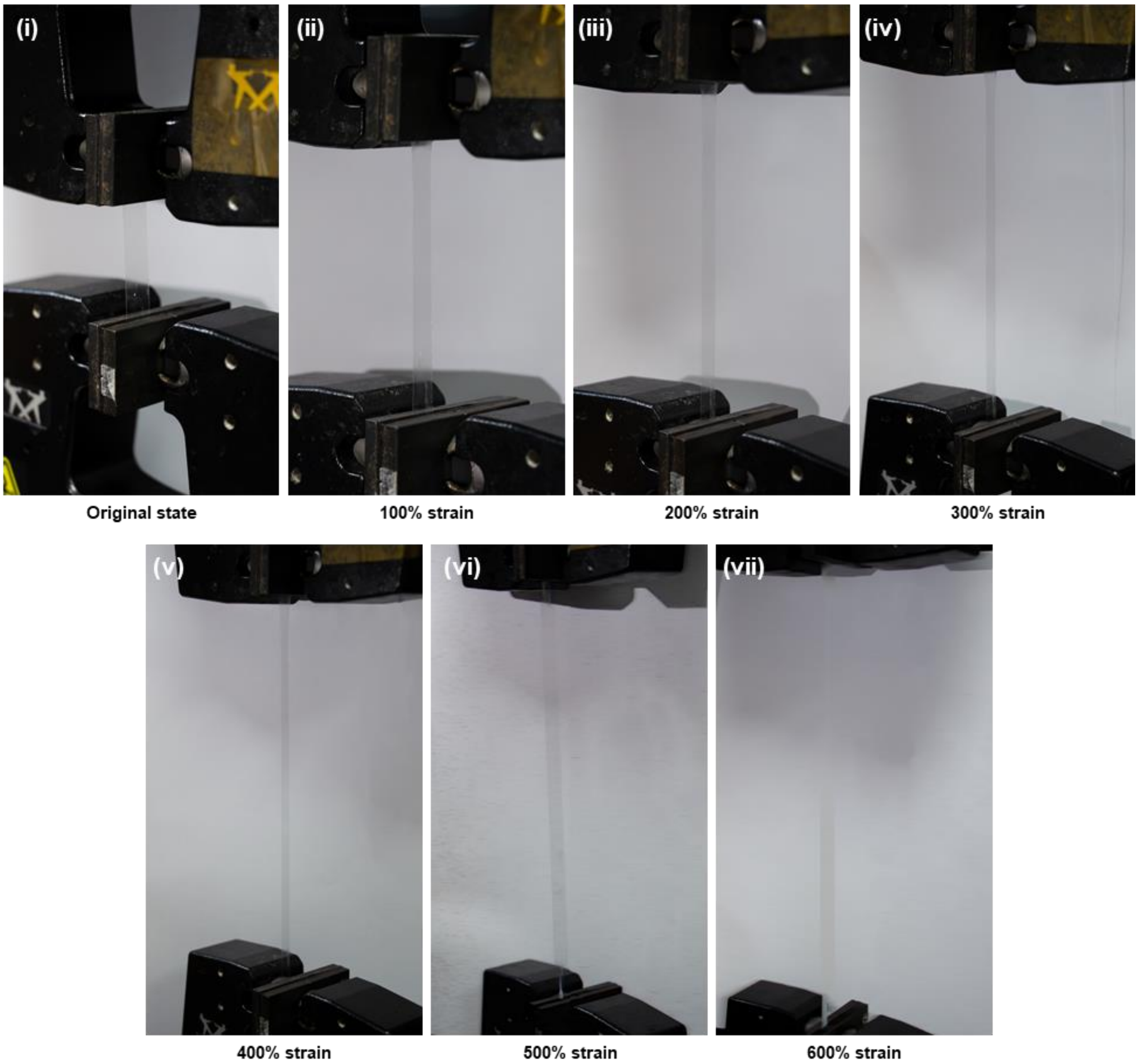

Figure S5. The deformation photographs of soft PU/double-side adhesive composite film of the electrode array under various uniaxial tensile strains.

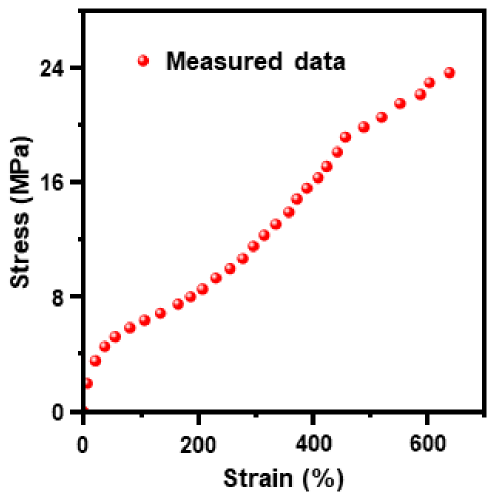

Figure S6. The measured stress-strain curve of soft PU/double-side adhesive composite film of the electrode array.

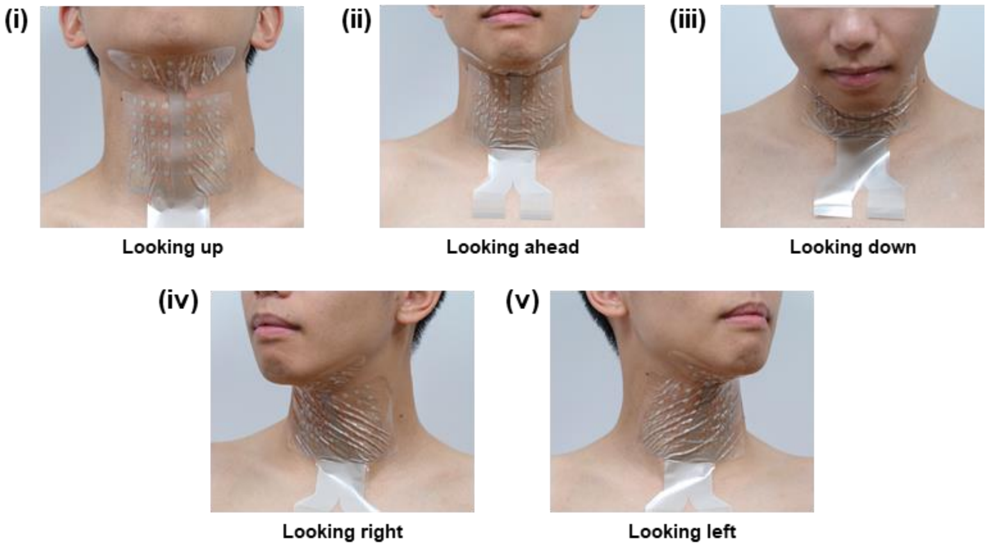

Figure S7. The deformed photographs of the electrode array adhered on the neck skin under five head movements. (A)

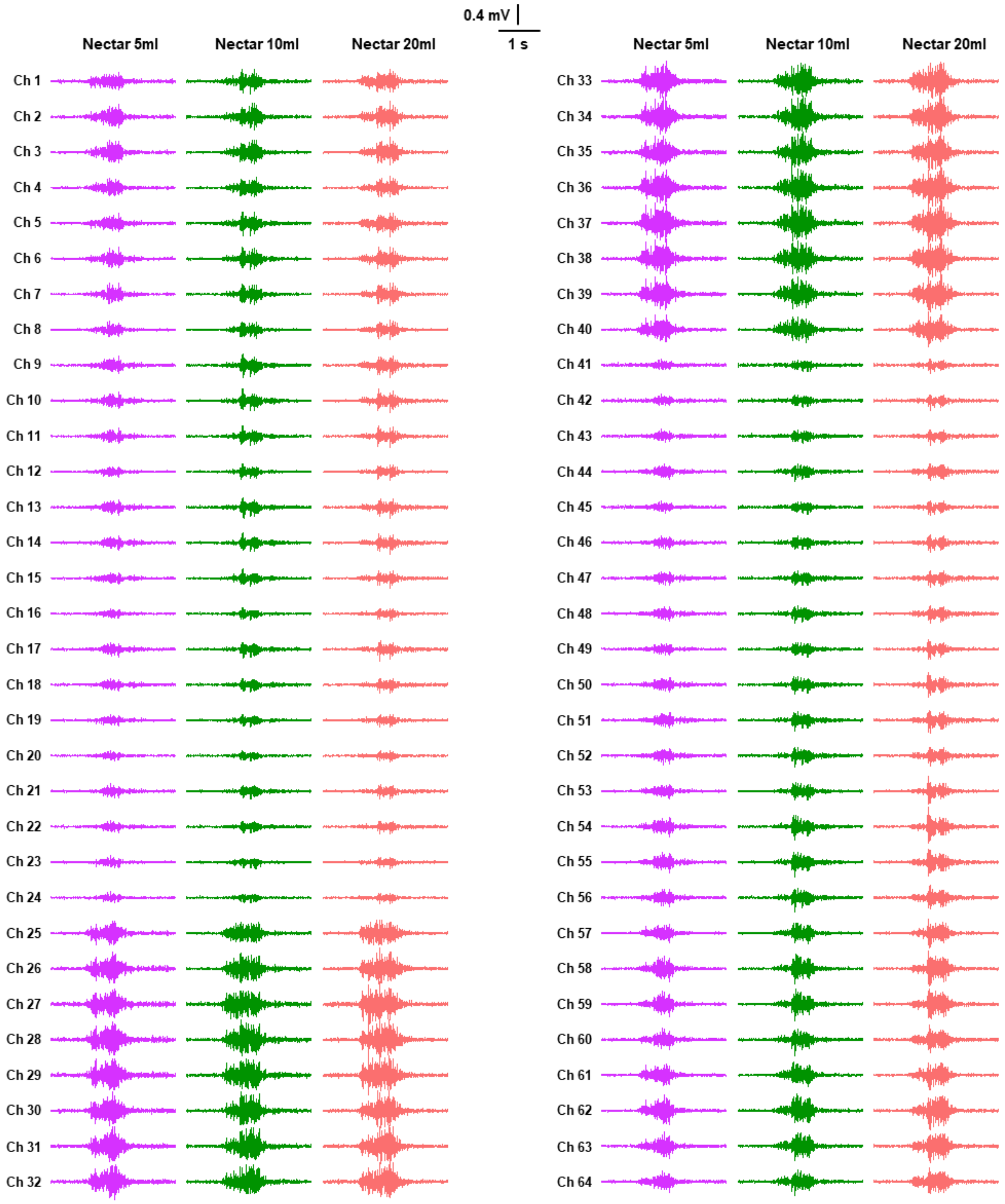

Figure S8. The collected 64-channel HD-sEMG signals from a participant during swallowing of 5 ml, 10 ml, and 20 ml of Nectar, respectively.

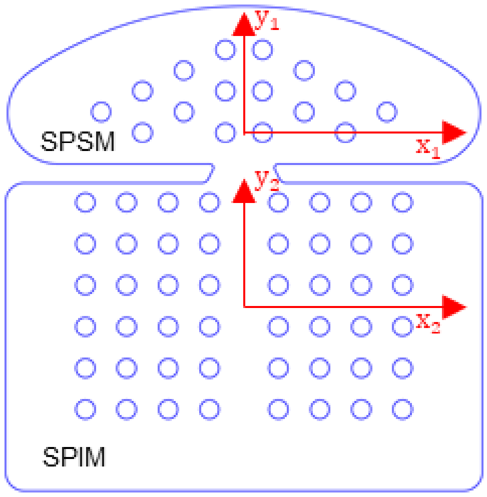

Figure S9. The x and y axes in the coordinate system for calculating the barycenter indicators B_x and B_y for the submental and infrahyoid muscles.

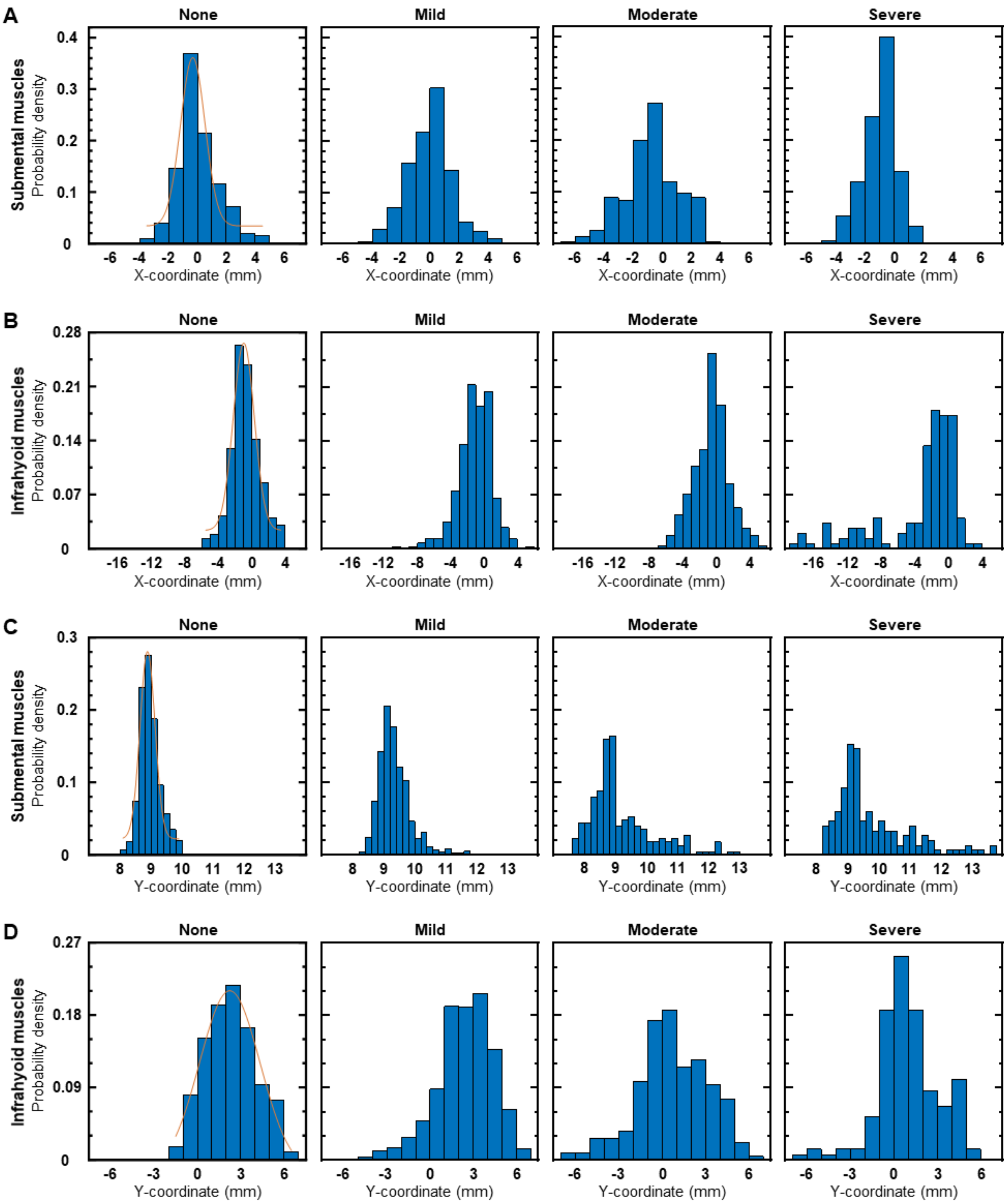

Figure S10. **The probability density distribution of the barycenter for the four types of participants.** The probability density distribution of the barycenter of muscle activity along the *x*-coordinate of (**A**) the submental muscles and (**B**) the infrahyoid muscles. The probability density distribution of the barycenter of muscle activity along the *y*-coordinate of (C) the submental muscles and (**D**) the infrahyoid muscles.

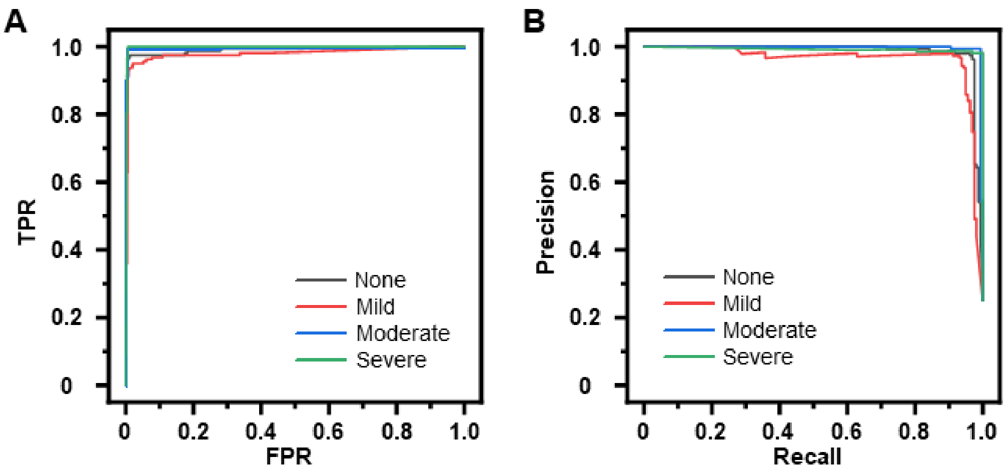

Figure S11. **(A)** the receiver operating characteristic curve and (**B**) the precision rate-recall rate curve of the dysphagia severity classification model.

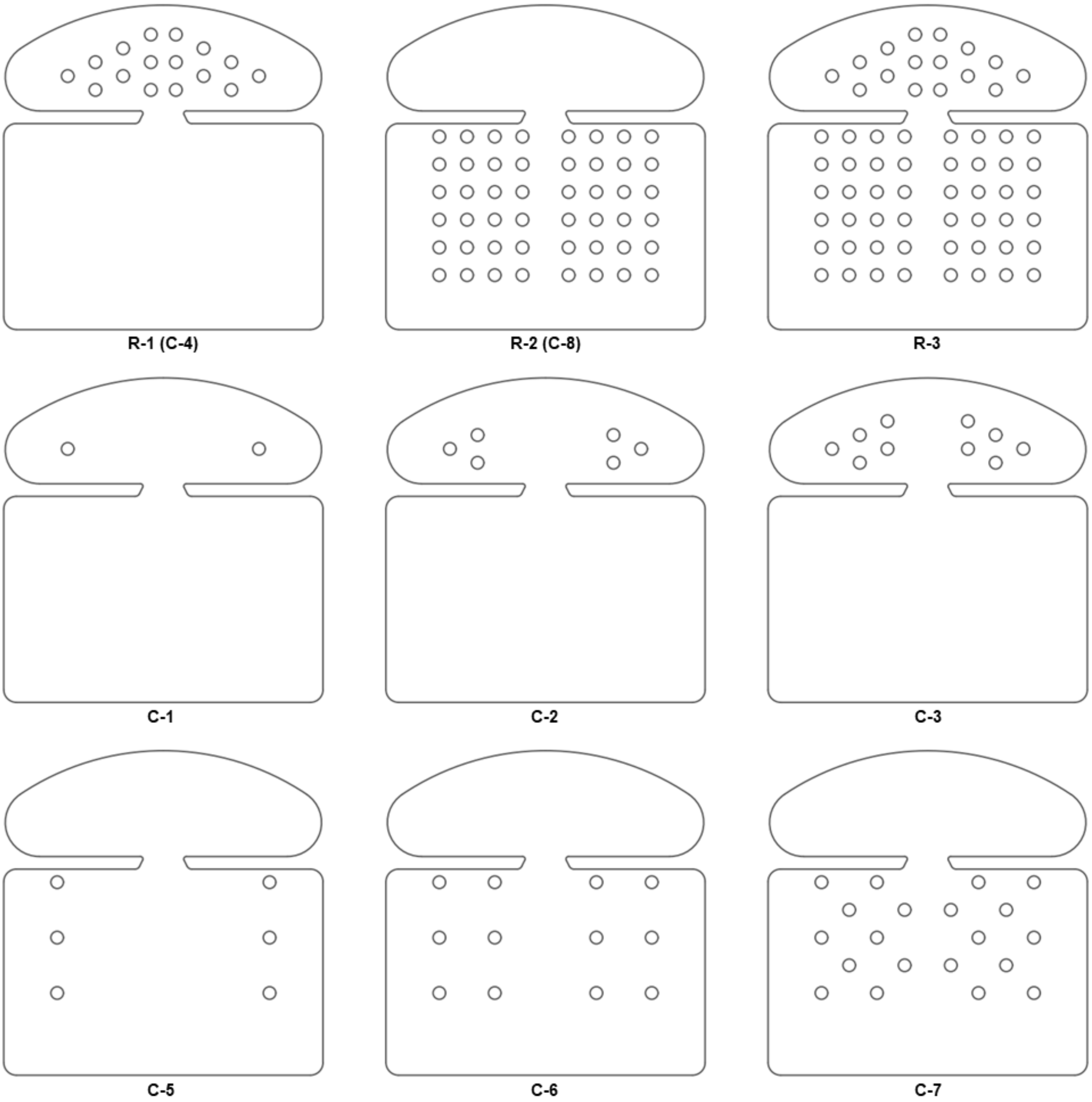

Figure S12. Various electrode distributions of the electrode array to investigate the effect of the electrode layout on the recognition accuracy of the model.

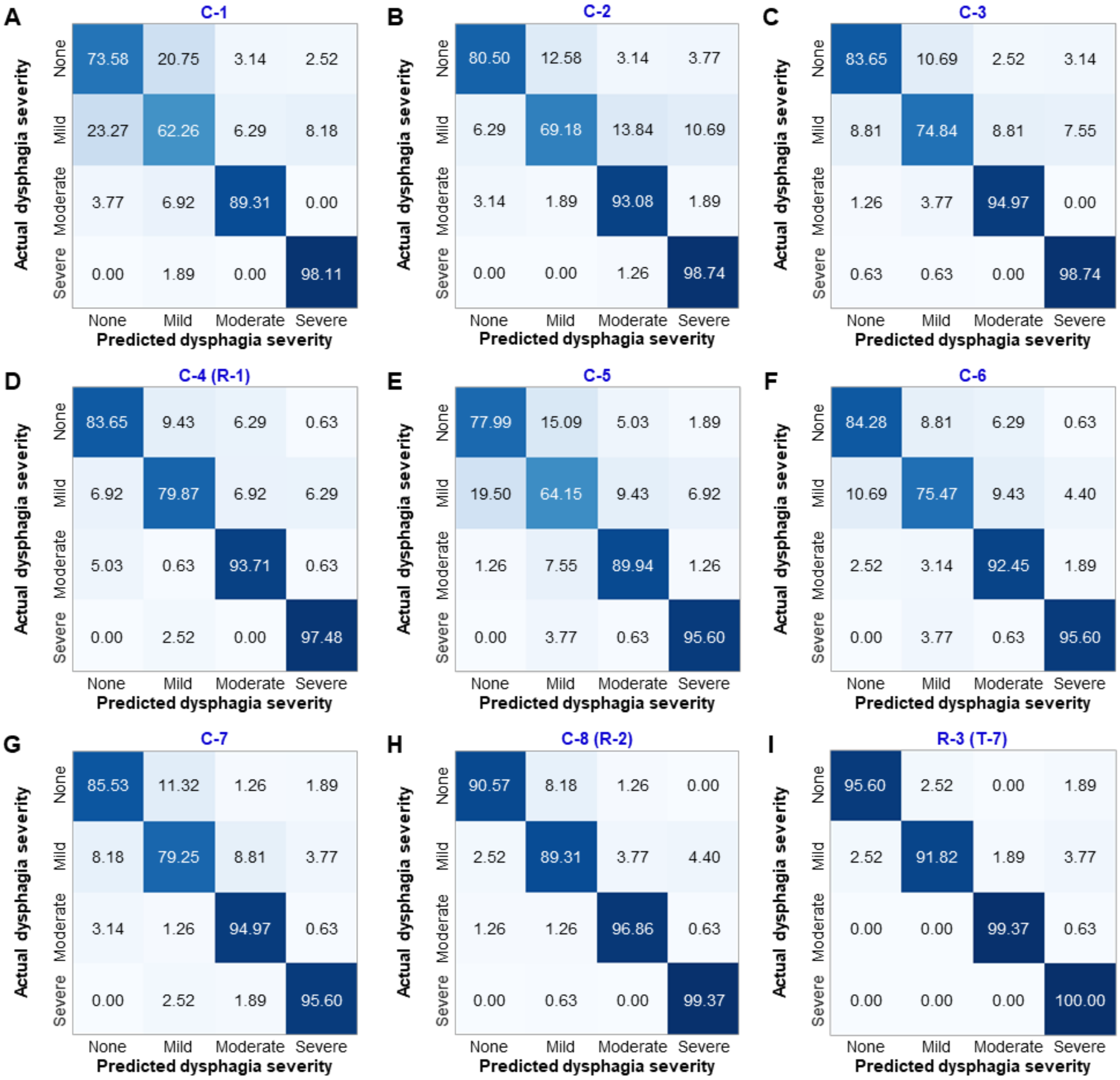

Figure S13. The calculated confusion matrixes of the dysphagia severity classification model under different electrode configurations.

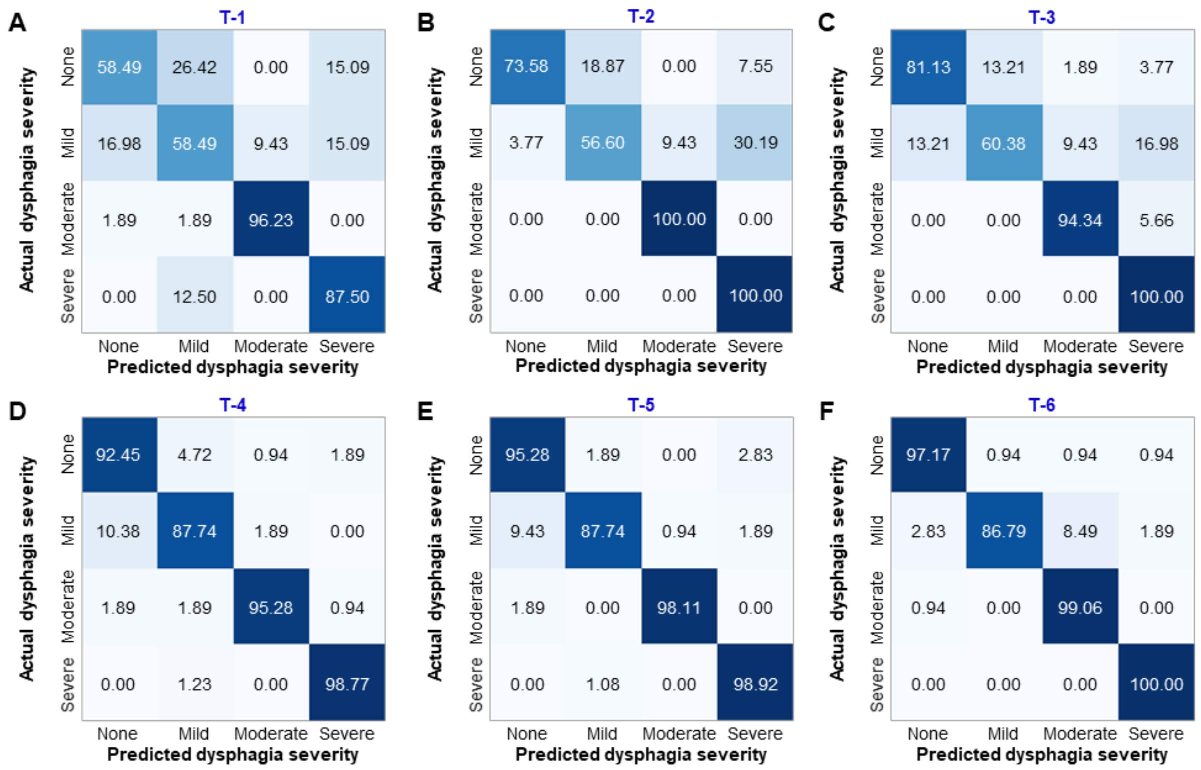

Figure S14. The calculated confusion matrixes of the dysphagia severity classification model under swallowing different type of food pellets.

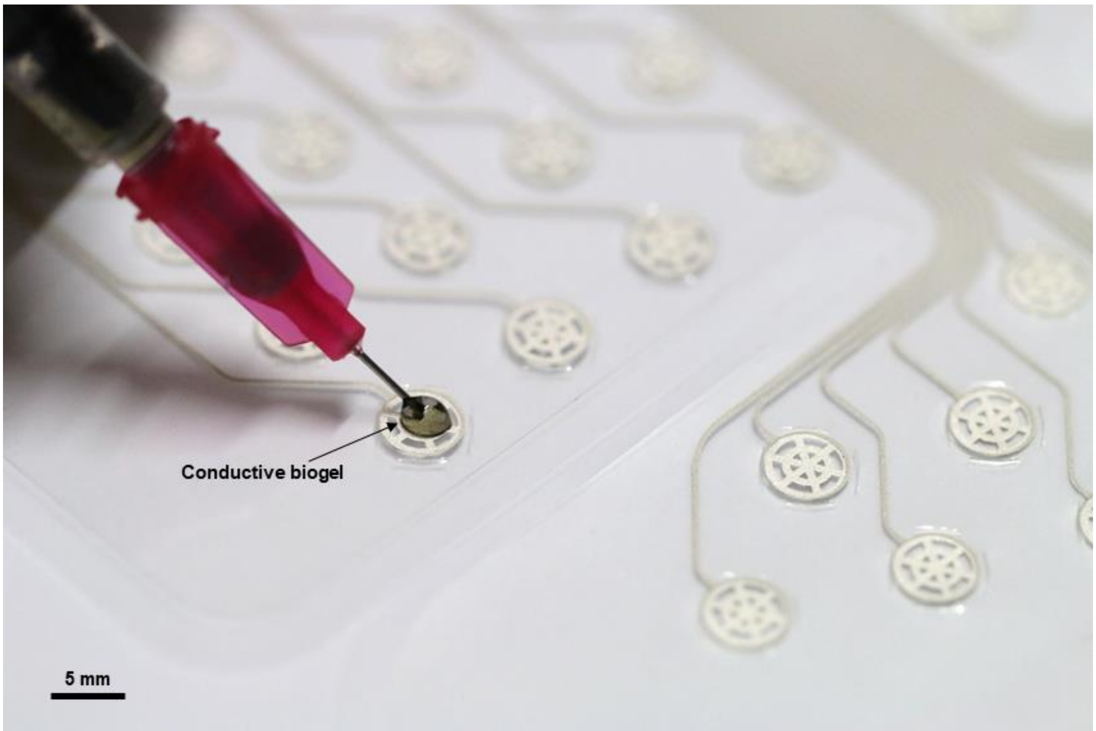

Figure S15. The integration of the tough, stretchable conductive biogel with the electrode array.

Table S1: The detailed information of the recruited participants.

Dysphagia severity	Male	Female	Total	Age	Cough
None	9	3	12	23.4 ± 2.2	
Mild	6	8	14	57.9 ± 3.7	
Moderate	5	2	7	70.1 ± 14.0	No
Severe	5	0	5	67.4 ± 13.1	Yes

Table S2: The detailed swallowing tasks for the four groups of participants.

Task	Dysphagia severity			
	None	Mild	Moderate	Severe
Nectar 5ml	37	58	15	9
Nectar 10ml	41	53	15	10
Nectar 20ml	36	49	13	8
Liquid 5ml	36	55	22	12
Liquid 10ml	41	58	13	0
Liquid 20ml	37	56	9	0
Pudding 5ml	38	59	14	13
Pudding 10ml	39	51	13	10
Pudding 20ml	36	50	15	10
Total	341	489	129	72

Table S3: The selected seven pellets to investigate the effect of the type of food pellets on the recognition accuracy of the model.

T-1	T-2	T-3	T-4	T-5	T-6	T-7
Liquid	Nectar	Pudding	Liquid + Pudding	Liquid + Nectar	Nectar + Pudding	All
